# RDH10 function is necessary for spontaneous fetal mouth movement that facilitates palate shelf elevation

**DOI:** 10.1242/dmm.039073

**Published:** 2019-07-03

**Authors:** Regina M. Friedl, Swetha Raja, Melissa A. Metzler, Niti D. Patel, Kenneth R. Brittian, Steven P. Jones, Lisa L. Sandell

**Affiliations:** 1 Department of Oral Immunology and Infectious Diseases, University of Louisville School of Dentistry, Louisville, KY 40202, USA; 2 Department of Medicine, Diabetes and Obesity Center, University of Louisville, Louisville, KY 40202, USA

**Keywords:** Retinoic acid, Cleft palate, Spontaneous mouth movement, RDH10, Vitamin A

## Abstract

Cleft palate is a common birth defect, occurring in approximately 1 in 1000 live births worldwide. Known etiological mechanisms of cleft palate include defects within developing palate shelf tissues, defects in mandibular growth and defects in spontaneous fetal mouth movement. Until now, experimental studies directly documenting fetal mouth immobility as an underlying cause of cleft palate have been limited to models lacking neurotransmission. This study extends the range of anomalies directly demonstrated to have fetal mouth movement defects correlated with cleft palate. Here, we show that mouse embryos deficient in retinoic acid (RA) have mispatterned pharyngeal nerves and skeletal elements that block spontaneous fetal mouth movement *in utero*. Using X-ray microtomography, *in utero* ultrasound video, *ex vivo* culture and tissue staining, we demonstrate that proper retinoid signaling and pharyngeal patterning are crucial for the fetal mouth movement needed for palate formation. Embryos with deficient retinoid signaling were generated by stage-specific inactivation of retinol dehydrogenase 10 (*Rdh10*), a gene crucial for the production of RA during embryogenesis. The finding that cleft palate in retinoid deficiency results from a lack of fetal mouth movement might help elucidate cleft palate etiology and improve early diagnosis in human disorders involving defects of pharyngeal development.

## Abstract (precis)

**Summary:** Fetal mouth immobility and defects in pharyngeal patterning underlie cleft palate in retinoid-deficient *Rdh10* mutant mouse embryos.

## INTRODUCTION

Orofacial clefts are among the most common human structural birth defects. Cleft lip/palate occurs at a prevalence of approximately 1 in 1000 live births worldwide, imposing a physical, social and fiscal burden on individuals, families and society (Parker et al., 2010; Wehby and Cassell, 2010; World Health Organization, 2007). The high incidence of palate clefts reflects the complexity of the process of palate formation during embryogenesis. Formation of the secondary palate involves initial downward vertical growth of the palate shelves, followed by tissue remodeling and horizontal growth of the shelves towards the midline, midline contact and finally fusion of the shelves by degradation of the midline epithelial seam (Bush and Jiang, 2012; Sperber, 2002).

Palate closure does not occur in isolation, but within the context of the many simultaneous morphogenic events occurring within a developing embryo. Concomitant with palate shelf elevation and medial growth, growth and expansion of the entire facial complex results in downward displacement of the mandible and tongue, opening space above in which the palate shelves elevate. The important morphogenic interplay between the growing mandibular and maxillary orofacial structures can be appreciated by considering the occurrence of cleft palate in Pierre Robin sequence. In this disorder, the primary etiology is underdevelopment of the mandible, with cleft palate occurring secondarily as a result of the tongue being crowded and obstructing palate shelf elevation (Giudice et al., 2018). The morphogenic events of orofacial growth and palate closure occur between 8 and 12 weeks gestation in humans (Burdi and Faist, 1967; Sperber, 2002; Yoon et al., 2000), corresponding to embryonic day (E) 12.5 to E16.5 in mouse (Bush and Jiang, 2012). Additional developmental events occurring at these stages include growth and targeting of motor and sensory nerves, pharyngeal skeletal development, and morphogenesis and activation of oropharyngeal musculature.

Concomitant with the growth and morphogenesis of orofacial anatomy, spontaneous neuromuscular movement of the fetal mandible and tongue begins (de Vries et al., 1985; Walker, 1969; Wragg et al., 1972). The spontaneous movements include a backwards tilt of the head away from the chest, opening of the mandible, and retraction and protrusion of the tongue. These movements coordinate the neuromuscular actions of swallowing amniotic fluid and presumably prepare the fetus for suckling after birth. The coordinated movements of swallowing require many anatomical elements including muscles, bones and cartilages, as well as innervation of motor nerves. Key skeletal elements include the hyoid bone, along with the thyroid and cricoid cartilages. These elements serve as anchoring attachments necessary for tongue and pharyngeal muscle function. Crucial motor nerves include the hypoglossal motor nerve (CN XII), which controls the tongue, and cervical nerve 1 (C1), which innervates the geniohyoid muscle that moves the hyoid bone.

It has long been speculated that movement of the mouth and tongue in developing fetuses facilitates palate formation (His, 1901). Evidence that functional tongue musculoskeletal anatomy is required for palate formation comes from analysis of *Hoxa2* (homeobox protein Hox-A2) mutant mouse embryos, in which defective muscle attachment correlates with cleft palate (Barrow and Capecchi, 1999). In addition to functional musculoskeletal anatomy, it is clear that functional neurotransmission is required for palate formation (Asada et al., 1997; Condie et al., 1997; Culiat et al., 1995; Homanics et al., 1997; Oh et al., 2010; Wojcik et al., 2006). These studies linking cleft palate phenotypes with defects in musculoskeletal anatomy or neurotransmission strongly suggest that the underlying etiology of clefting in these experimental models is fetal mouth immobility. Ultrasound monitoring of fetal mouth movement *in utero* has directly demonstrated that the cleft palate of mouse fetuses deficient in the inhibitory neurotransmitter γ-aminobutyric acid is correlated with lack of fetal mouth movement (Tsunekawa et al., 2005). Fetal mouth immobility correlating with cleft palate has also been documented directly by ultrasound in a human fetus with a syngnathic jaw fusion (Laster et al., 2001).

Whereas studies directly documenting impaired mouth movement *in utero* are rare, there is ample evidence for defects in swallowing movements during postnatal life. Pediatric dysphagia, difficulty swallowing and feeding that begins at birth, is a common human disorder (LaMantia et al., 2016; Miller, 2009). It is a key phenotype of human 22q11.2 deletion syndrome, also known as DiGeorge syndrome, which is characterized by a spectrum of abnormalities including cleft palate, orofacial malformations and defects in development of the pharyngeal arch derivatives (LaMantia et al., 2016; McDonald-McGinn et al., 2015). Comparison of distinct mutant mouse models of 22q11.2 deletion syndrome has helped to elucidate the pathogenic mechanisms underlying the postnatal dysphagia phenotype, and has shown that swallowing difficulties can be attributed to loss of function of CN XII (Karpinski et al., 2014; Wang et al., 2017).

The nerves, bones and cartilages that work synergistically in swallowing are derived from the pharyngeal arches during embryonic development. The second to sixth pharyngeal arches develop into fundamental components of the head and neck that are crucial for swallowing. It is well established that pharyngeal arch development is orchestrated by retinoic acid (RA) signaling, and that perturbation of RA levels results in defects of the pharyngeal-arch-derived elements (Mark et al., 2004; Wendling et al., 2000).

RA is the active metabolite of the dietary small molecule vitamin A. Collectively, vitamin A and its derivatives are known as retinoids. The metabolic conversion of vitamin A to RA is accomplished through two sequential oxidative reactions (Duester, 2008; Niederreither and Dollé, 2008), the first of which is mediated in embryos primarily by the enzyme retinol dehydrogenase 10 (RDH10) (Sandell et al., 2012a, 2007). RA is an important signaling molecule that regulates many aspects of adult health and embryonic development. One developmental role of RA that has been extensively studied is the regulation of embryonic anterior-posterior patterning, which occurs largely through the transcriptional regulation of *Hox* gene family members (Halilagic et al., 2007, 2003; Hernandez et al., 2007; Rhinn and Dolle, 2012; Ribes et al., 2006; Schneider et al., 2001; Sirbu et al., 2005). Homeostatic levels of RA must be tightly controlled within embryonic tissues, as excess or insufficiency both disrupt embryogenesis (Cunningham and Duester, 2015; Metzler and Sandell, 2016; Niederreither and Dollé, 2008).

Many regions of the embryo are sensitive to disturbances in RA signaling. Malformations resulting from RA perturbation include defects in development of the pharyngeal arch derivatives (Mark et al., 2004; Wendling et al., 2000) and cleft secondary palate (Hale, 1935; Halilagic et al., 2007; Lohnes et al., 1994; Sandell et al., 2007; Warkany, 1945; Warkany et al., 1943; Wilson et al., 1953). The underlying mechanisms of cleft palate resulting from perturbations of RA signaling remain unclear. Mouse embryos with excess RA signaling resulting from deficient degradation have been shown to have abnormal tongue height and defects in the proliferation of cells in the bend region of the palate shelves (Okano et al., 2012). Excess RA, resulting either from deficient degradation or teratogenic overexposure, is said to induce cleft palate by mechanisms such as arresting the cell cycle in mesenchymal cells of palate shelves, causing increased apoptosis or through abnormal cell differentiation in the midline epithelial edge (Hu et al., 2013; Huang et al., 2003; Nelson et al., 2011; Okano et al., 2007). Thus, there are multiple, and in some cases conflicting, mechanisms proposed to explain the formation of cleft palate under conditions of RA excess. By contrast, the developmental etiology of cleft palate in conditions of RA deficiency is still unknown.

Here, using conditional *Rdh10* mutant mice to investigate the etiology of cleft palate in retinoid deficiency, we identify a link between retinoid signaling, pharyngeal neuroskeletal morphogenesis, fetal mouth movement and palate formation.

## RESULTS

### Stage-specific inactivation of *Rdh10* induces secondary cleft palate

Vitamin A metabolism and RA production are essential for viability in the early organogenesis stages of development; severe deficiency of RA signaling from inception can result in embryonic lethality before palate morphogenesis (Niederreither et al., 1999; Sandell et al., 2012b; See et al., 2008; White et al., 1998). Therefore, in order to understand the requirement for vitamin A metabolism and RA signaling during palate development, we reduced embryonic production of RA after the onset of RA signaling by inducing inactivation of *Rdh10*, a gene crucial for RA production during embryogenesis (Sandell et al., 2012b, 2007). Reduction of RA signaling was accomplished by stage-specific inactivation of a conditional floxed allele of *Rdh10* (Sandell et al., 2012b) by Cre–mediated excision via the tamoxifen-inducible Cre-ERT2 (Ventura et al., 2007).

The *Rdh10* alleles used in this study include *Rdh10^+^*, which denotes the wild-type allele, *Rdh10^delta^*, which denotes a targeted knockout null allele with exon 2 deleted, and *Rdh10^flox^*, a floxed allele in which exon 2 is excised upon exposure to Cre recombinase, thereby converting to *Rdh10^delta^* (Sandell et al., 2012b). Disruption of RA production at different embryonic stages can produce a variety of phenotypes (See et al., 2008; White et al., 1998). In a previous study, *Rdh10* was conditionally eliminated by Cre-ERT2 with tamoxifen administered at E7.5 to study the role of RA signaling in nasal airway development (Kurosaka et al., 2017). In the current study, we utilize the same mouse strains to conditionally inactivate RDH10 function by administration of tamoxifen at E8.5. These experimental conditions, with tamoxifen administered at E8.5, have been previously validated to completely eliminate *Rdh10* RNA by E10.5, which attenuates RA signaling activity to 30% that of control embryos by E11.5 (Metzler et al., 2018). It is important to note that, using the ubiquitously expressed Cre-ERT2, the genotype of embryos with a floxed allele changes following administration of tamoxifen. Embryos with a pre-tamoxifen genotype of *Rdh10^flox/+^* become *Rdh10^delta/+^* post–tamoxifen. Embryos with a pre-tamoxifen genotype of *Rdh10^delta/flox^* or *Rdh10^flox/flox^* become *Rdh10^delta/delta^* post-tamoxifen treatment. Throughout this study, all references to genotype will denote the pre-tamoxifen condition.

**Fig. 1.**
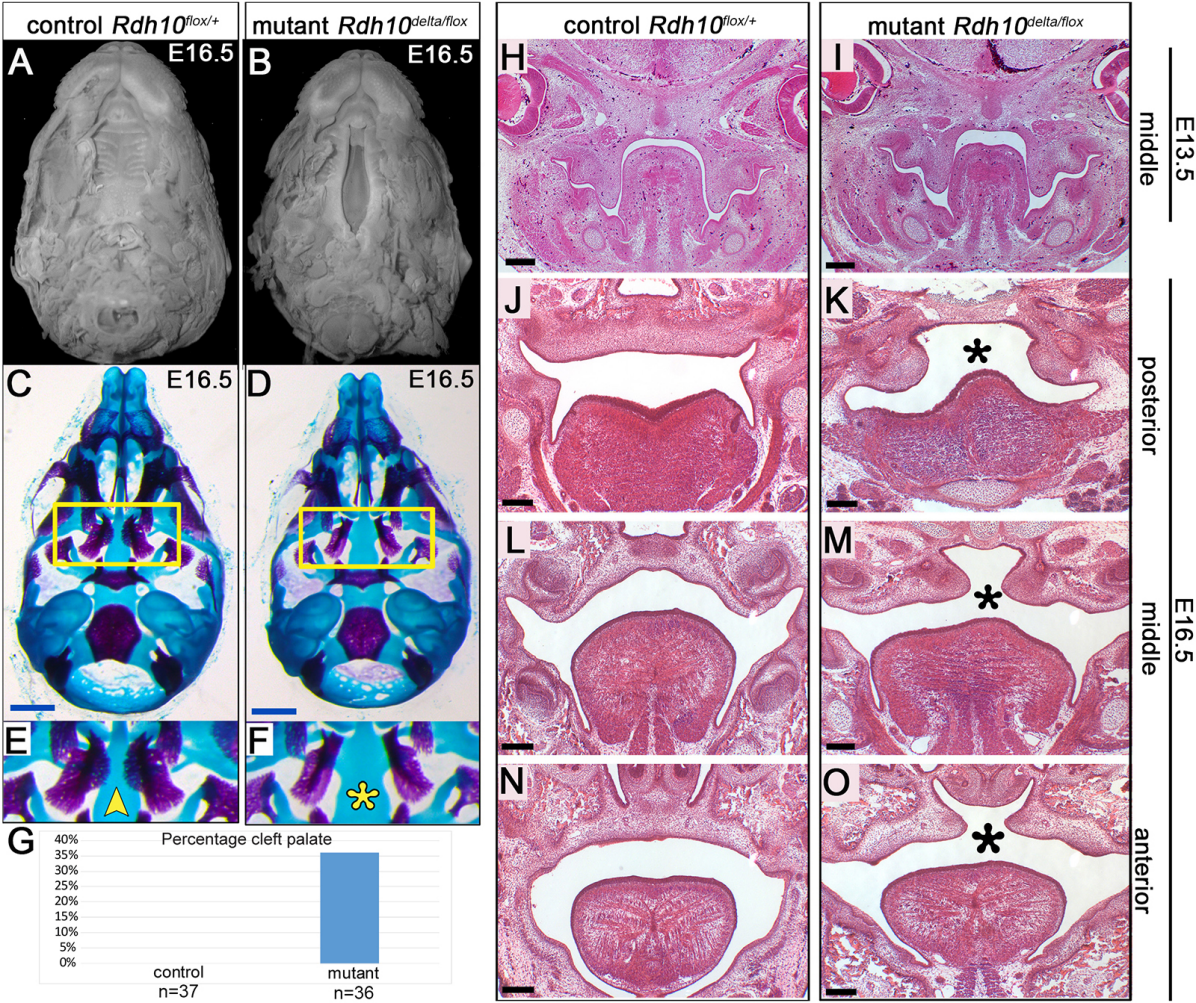
**Stage-specific inactivation of retinol metabolism in *Rdh10^delta/flox^* mutant embryos serves as a model for vitamin A/retinoid-deficient cleft palate.** Conditional inactivation of *Rdh10* causes cleft palate. Nuclear fluorescence imaging of *Rdh10^flox/+^* control (A) and *Rdh10^delta/flox^* mutant (B) embryos at E16.5 reveals complete cleft of the secondary palate in 36% of mutant embryos (G). (C-F) Bone and cartilage staining with Alizarin Red and Alcian Blue of E16.5 embryos. Palatine bones of control embryos have grown towards the midline with feathering outgrowths (C,E yellow arrowhead) (*n*=15/15). By contrast, palatine bones of a subset of *Rdh10^delta/flox^* mutant embryos remain lateral with no medial growth of bone towards the midline (D,F yellow asterisk) (*n*=7/11). The *Rdh10^delta/flox^* conditional inactivation model produces cleft palate at a frequency of 36% at E16.5 (G), which is significant based on the Fisher's exact test for independence. Midpalate coronal sections stained with H&E reveal that (H) control and (I) mutant specimens are similar at E13.5, with palate shelves vertically oriented on either side of the tongue. (J-O) H&E stained sections of E16.5 embryos reveal the cleft palate defect in mutant embryos. At this stage, palate shelves of control embryos have elevated, grown towards the midline and fused in the posterior (J), middle (L) and anterior (N) palate. By contrast, palate shelves of a subset of mutant embryos are open and unfused in the posterior (K), middle (M) and anterior (O) palate. Black asterisks denote lack of medial contact of mutant palate shelves. (C,D) Blue scale bars: 1 mm. (H-O) Black scale bars: 200 µm.

To determine if RDH10 and endogenous RA are important for secondary palate formation, we assessed palate morphology in *Rdh10^flox/+^* control and *Rdh10^delta/flox^* mutant embryos at E16.5. Palates of embryos, with mandibles removed, were visualized by nuclear fluorescence staining (Fig. 1A,B). Cleft of the secondary palate was observed in 36% of *Rdh10^delta/flox^* mutant embryos (Fig. 1B,G; *n*=36). By contrast, cleft palate was not observed in any *Rdh10^flox/+^* control embryos (Fig. 1A,G; *n*=37).

Overall cranial morphology of the *Rdh10^delta/flox^* mutant embryos resembled that of *Rdh10^flox/+^* control littermates. Bone and cartilage staining of E16.5 embryos revealed that most of the cranial skeletal elements were present in mutant embryos (Fig. 1C,D). However, defects in palatine bone morphogenesis, consistent with the palate cleft observed in whole-mount specimens, were observed in a subset of skeletal-stained mutant embryos (Fig. 1C-F). In all control embryos the two opposing palatine bones had a feathery outgrowth that almost touched at the midline (Fig. 1C,E, yellow arrow; *n*=15/15), consistent with normal palate closure. By contrast, in 50% of *Rdh10^delta/flox^* mutant skulls, the feathery medial growth of the palatine bones was lacking and the ossified palatine bones did not approach the midline (Fig. 1D,F yellow asterisks; *n*=7/11), consistent with cleft palate.

To gain insight into the tissue architecture in cleft palates of *Rdh10^delta/flox^* mutant embryos, we performed Hematoxylin and eosin (H&E) staining of paraffin sections. At E13.5, the palate shelf morphology of *Rdh10^delta/flox^* mutant embryos resembled that of *Rdh10^flox/+^* control littermates, with palate shelves aligned vertically on either side of the tongue (Fig. 1H,I). At E14.5, the stage when palate shelf re-orientation occurs, 82% of *Rdh10^flox/+^* control embryos had both palate shelves oriented horizontally, whereas only 33% of mutant embryos had both shelves in the horizontal orientation (Fig. S1). At this stage unilateral palate shelf elevation was observed at low frequency in both genotypes (Fig. S1). By E16.5, the palate shelves of *Rdh10^flox/+^* control embryos have elevated and fused at the midline (Fig. 1J,L,N). By contrast, the palate shelves of ∼40% of *Rdh10^delta/flox^* mutant embryos appear elevated, but have not grown towards the midline (Fig. 1K,M,O). Together, the histological, whole-mount and skeletal data reveal that *Rdh10^delta/flox^* mutant embryos administered tamoxifen at E8.5 have reproducible cleft palate, demonstrating that this conditional inactivation model can be used to study the role of endogenous vitamin A and RA signaling in palate development.

### The underlying cause of cleft palate in retinoid-deficient embryos is extrinsic to palate shelves, but mandible size is not reduced

**Fig. 2.**
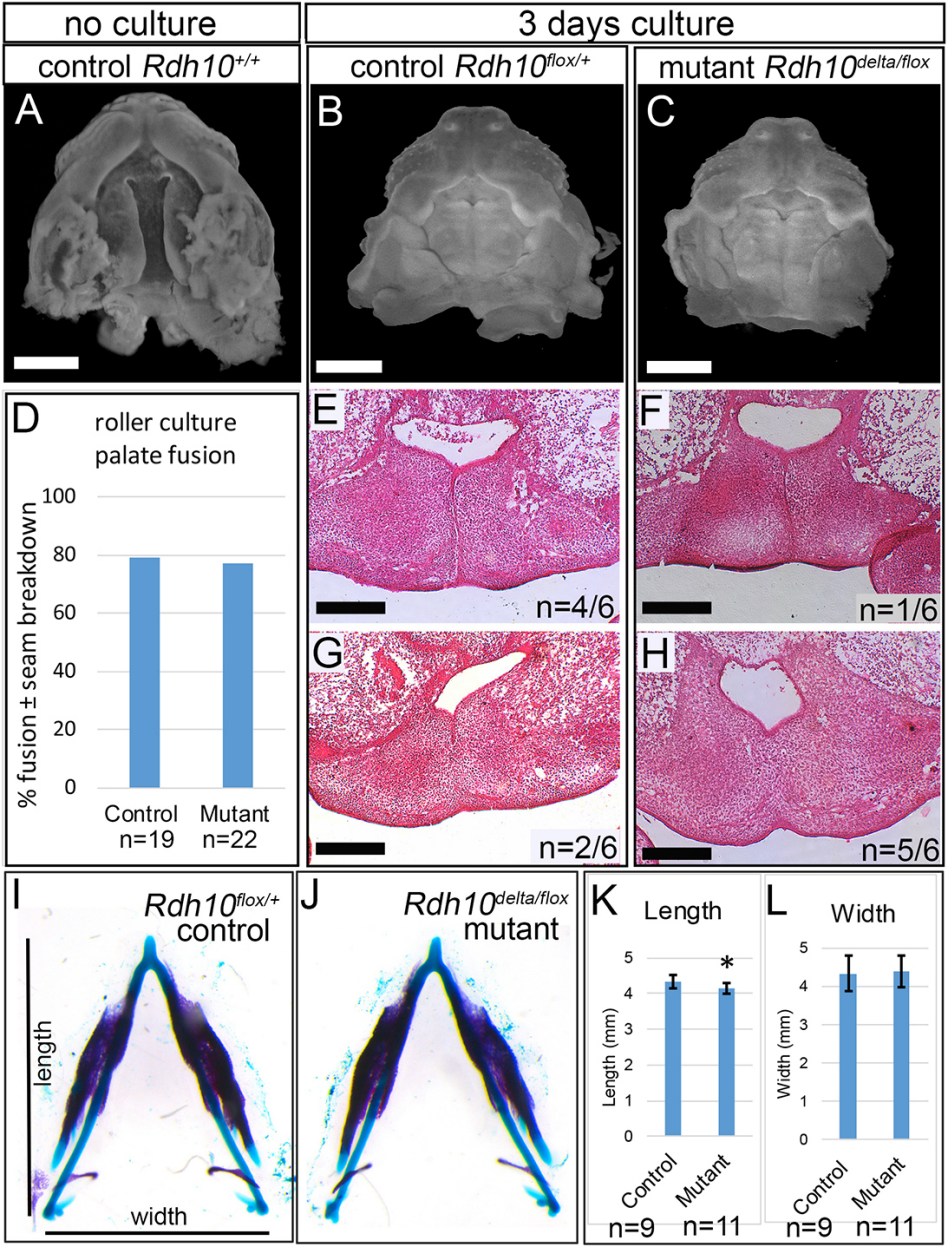
***Rdh10^delta/flox^* mutant embryos have palate shelves that elevate and fuse when cultured *ex vivo*, but do not have micrognathia.** (A-C) Maxillary explants visualized by nuclear fluorescence imaging. (A) E13.5 maxillary explants with unfused palate shelves were dissected free of brain, mandible and tongue prior to *ex vivo* suspension culture. (B,C) After 72 h in suspension culture, both *Rdh10^flox/+^* control (B) and *Rdh10^delta/flox^* mutant (C) embryos exhibit apparent fusion of palate shelves. (D) The frequency of apparent fusion for control and mutant explants is similar (control *n*=19, mutant *n*=22). The χ^2^ test for independence indicates no significant difference between control and mutant explants; *P*≥0.05. (E-H) H&E staining of coronal sections through the cultured maxillae reveals complete fusion with breakdown of midline epithelial seam in a subset of control and mutant specimens. For sectioned control specimens 4/6 retained the midline epithelial seam (E), whereas 2/6 had evidence of loss of epithelial seam indicating palate fusion (G). For sectioned mutant specimens 1/6 retained the midline epithelial seam (F), whereas 5/6 had evidence of fusion and loss of epithelial seam (H). (I,J) Mandibles were isolated from E16.5 *Rdh10^flox/+^* control (*n*=9) and *Rdh10^delta/flox^* mutant embryos (*n*=11) and stained with Alcian Blue and Alizarin Red to reveal bone and cartilage. Stained mandibles were imaged, measured for length and width and measurements were compared for mutant versus control embryos within litters. The length of the mandibles of mutant embryos was slightly shorter than that of control littermates (K); *P=0.01. No difference in width was detected between mutants and controls (L). The significance of intralitter comparison from multiple litters was assessed by *t*-test using a linear mixed effects model with litter as the random effect. Error bars represent standard error of the mean. (A-C) White scale bars: 1 mm. (E-H) Black scale bars: 200 µm.

Cleft palate can be caused by defects intrinsic to the palate shelves or by defects in other tissues that indirectly prevent palate closure. To determine whether cleft palate in retinoid deficiency occurs by a mechanism intrinsic or extrinsic to the palate shelves, we assessed the fusion of maxillary explants cultured independently of the tongue and mandible. Maxillary tissues were isolated from E13.5 embryos, before shelf elevation (Fig. 2A), and were placed in a rolling suspension culture for 72 h to allow horizontal re-orientation and fusion to take place (Lan et al., 2016). Under these conditions, palate shelf elevation and medial contact occurred in 79% of the maxillary explants from *Rdh10^flox/+^* control embryos (Fig. 2B,D; *n*=19). Similarly, the maxillary explants from *Rdh10^delta/flox^* mutant embryos elevated and made medial contact at a rate of 77% (Fig. 2C,D; *n*=22). The two experimental groups are not statistically different (χ^2^, *P*>0.05). These data indicate that mutant maxillary tissues have no intrinsic defect in palate shelf elevation or medial growth.

To determine if fusion occurred in cultured maxillae, a subset of cultured maxillary explants were examined histologically (Fig. 2E-H). Complete fusion with breakdown of the midline epithelial seam was observed in both *Rdh10^flox/+^* control palate explants (*n*=2/6) and *Rdh10^delta/flox^* mutant palate explants (*n*=5/6). The occurrence of palate shelf elevation, medial growth and midline fusion in cultured maxillary explants of *Rdh10^delta/flox^* mutant embryos demonstrates that the underlying mechanism of cleft palate in these embryos is extrinsic to the palate shelves.

One known extrinsic defect that can cause cleft palate is micrognathia, in which the small mandible crowds the tongue in the back of the oral cavity preventing palate closure. To evaluate if retinoid deficiency causes micrognathia, we analyzed mandible size in *Rdh10^flox/+^* control and *Rdh10^delta/flox^* mutant embryos at E16.5 (Fig. 2I,J). After staining head specimens for bone and cartilage with Alizarin Red and Alcian Blue, mandibles were isolated by microdissection, imaged and mandible length and width were measured. Mandibles of *Rdh10^delta/flox^* mutant embryos were slightly (≤5%) shorter than *Rdh10^flox/+^* control littermates (Fig. 2K). No difference in mandible width was observed (Fig. 2L) and no obvious differences were noted in bone or cartilage morphology between the two groups. The similarity in size of mandibles in *Rdh10^delta/flox^* mutant embryos relative to *Rdh10^flox/+^* controls suggests that micrognathia is not the cause of cleft palate in retinoid deficiency.

### The tongue of *Rdh10^delta/flox^* mutant embryos obstructs palate shelf elevation

**Fig. 3.**
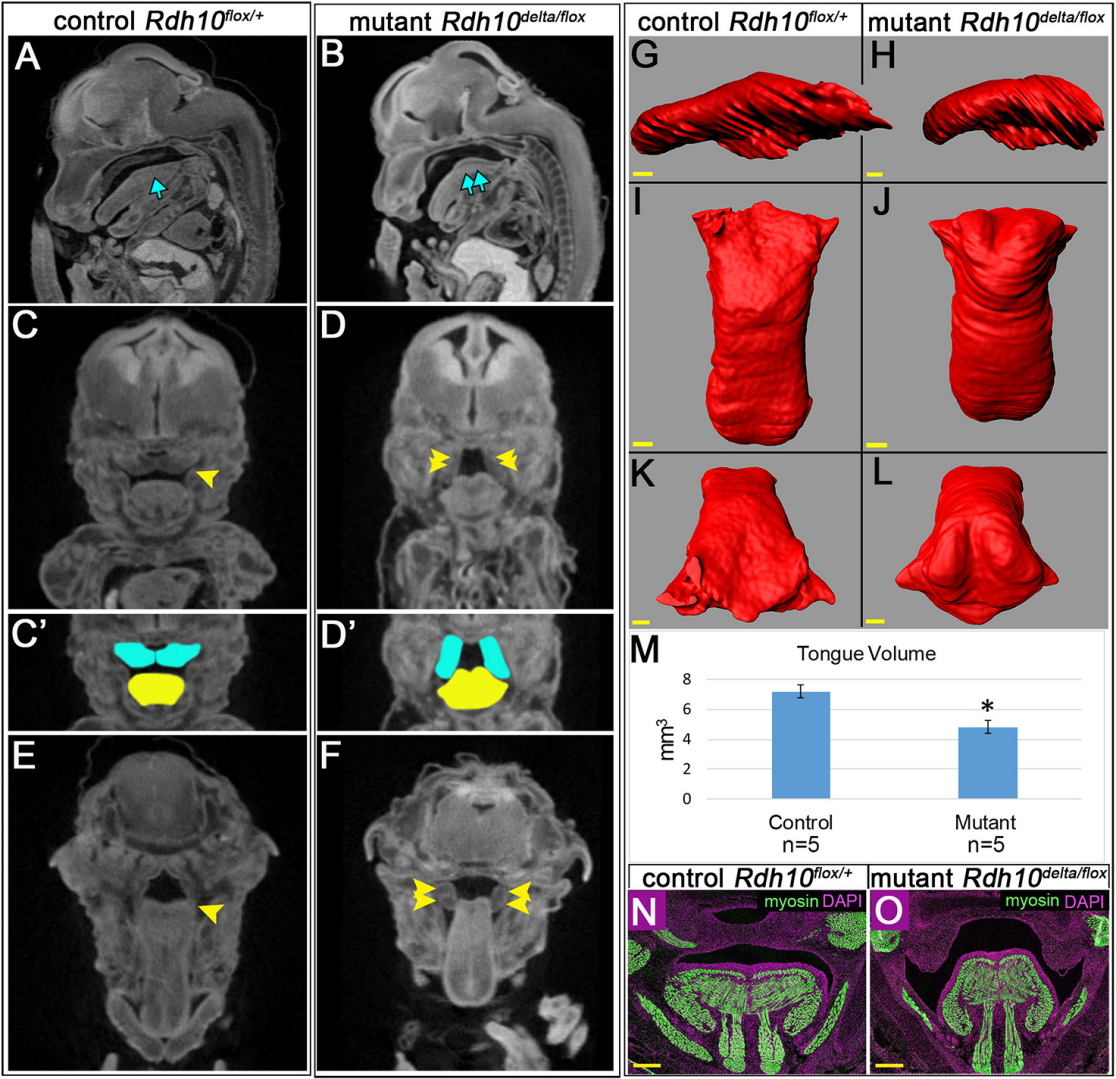
**Analysis of embryo morphology by microCT reveals *Rdh10^delta/flox^* mutants have abnormally positioned tongues that obstruct palate shelf elevation.** MicroCT scans of E14.5 *Rdh10^flox/+^* control (A,C,C′,E) and *Rdh10^delta/flox^* mutant (B,D,D′,F) embryos. Sagittal view at the midline shows the tongue of control embryo lies flat under the posterior palate shelf (A, single blue arrow, *n*=5/5), whereas the mutant embryo tongue is arched in the back of the oral cavity (B, double blue arrow, *n*=6/6) with no posterior palate shelf visible in the midsagittal plane. (C) Coronal view of control embryos shows that palate shelves have elevated over the tongue and contact at the midline (single yellow arrowhead, *n*=4/5 both shelves elevated, *n*=1/5 one shelf elevated). (D) By contrast, coronal view of mutant embryos reveals the palate shelves oriented vertically, appearing obstructed by the arched tongue (double yellow arrowhead, *n*=5/6). (C′) Color-coded image of (C) with blue palate shelves elevated over a yellow flattened tongue. (D′) Color-coded image of (D) with blue palate shelves trapped vertically on each side of the tongue. (E) Transverse section at the level just above the tongue reveals the control tongue has flattened out underneath the palate shelves that are elevated out of view (single yellow arrowhead). (F) Transverse section above the mutant tongue reveals the posterior palate shelves wedged laterally on either side of the tongue (double yellow arrowhead). Volume-rendering of the control (G,I,K) and mutant (H,J,L) tongues gives a sagittal (G,H), dorsal (I,J) and posterior view (K,L) of the tongue morphology. (M) The volumetric analysis shows the mutant tongues (*n*=5) are smaller in volume than control tongues (*n*=5); *P≤0.05 via Student's *t*-test. Immunofluorescence staining for myosin on E14.5 coronal sections of control (N, *n*=3) and mutant (O, *n*=5) tongues reveals that mutant tongue musculature is grossly normal. Scale bars: 200 µm.

To gain insight into the causative morphological defects preceding cleft palate in retinoid-deficient embryos, we performed X-ray microtomography (microCT) analysis on *Rdh10^flox/+^* control and *Rdh10^delta/flox^* mutant embryos at E14.5. Reconstructed 3D matrix files were rendered to reveal sagittal, coronal and transverse section images. Comparison of section views revealed a noticeable difference in tongue position for *Rdh10^delta/flox^* mutant embryos relative to *Rdh10^flox/+^* control littermates. In sagittal views, the tongues of control embryos appeared relatively flat (Fig. 3A, single blue arrow; *n*=5/5), whereas the tongues of mutant embryos were arched in the posterior (Fig. 3B, double blue arrows; *n*= 6/6). In the sagittal midline section views, control embryo palate shelves were visible along their length, indicating that the shelves had grown towards the midline, whereas in mutant embryos posterior palate shelves were not visible near the midline. Comparison of coronal sections revealed that the palate shelves of *Rdh10^flox/+^* control embryos were elevated horizontally above the flattened tongues (Fig. 3C,C′; *n*=4/5 both shelves elevated, *n*=1/5 one shelf elevated), whereas *Rdh10^delta/flox^* mutant embryos had vertical palate shelves positioned on either side of the arched tongue (Fig. 3D,D′; *n*=5/6 both shelves vertical, appearing obstructed by tongue, *n*=1/6 both shelves elevated). For control embryos, transverse sections at the level of the tongue revealed that the tongue was elongated and lying completely under the palate shelves, which were out of view (Fig. 3E). By contrast, in *Rdh10^delta/flox^* mutant embryos palate shelves appeared to be trapped by the tongue at the posterior end (Fig. 3F). Together, these data suggest that E14.5 *Rdh10^delta/flox^* mutant embryos have palate shelves apparently obstructed from elevating owing to malpositioning of the tongue.

To determine if tongue obstruction in the *Rdh10^delta/flox^* mutant embryos resulted from increased tongue volume, we measured tongue volume in control and mutant embryos by volume-rendering microCT data sets (Fig. 3G-L). Volumetric analysis showed that *Rdh10^delta/flox^* mutant tongues were not enlarged, but were instead slightly smaller in volume than the tongues of control littermates (Fig. 3M). These data indicate that tongue obstruction of the palate shelves in *Rdh10^delta/flox^* mutant embryos is not caused by increased tongue volume.

Our observation of the abnormal arched and contracted appearance of tongues in *Rdh10^delta/flox^* mutant embryos led us to evaluate the intrinsic tongue muscles of the tongue. We performed immunostaining for the muscle marker myosin on coronal sections of E14.5 *Rdh10^delta/flox^* mutant and *Rdh10^flox/+^* control embryos (Fig. 3N,O). No defect in the intrinsic tongue muscles was detected in mutant embryos relative to control littermates. All intrinsic muscles of the tongue, as well as the genioglossus fibers, appeared to be present and normal in the *Rdh10^delta/flox^* mutant embryos. Collectively, these results indicate the morphogenesis of tongue musculature is grossly normal in retinoid-deficient embryos, suggesting the abnormal tongue shape does not result from aberrant muscle morphogenesis.

### Spontaneous fetal mouth movement *in utero* is restricted in *Rdh10^delta/flox^* mutant embryos

The observation that *Rdh10^delta/flox^* mutant embryos had malpositioned tongues obstructing palate shelf elevation, in the context of sufficient-sized mandibles, led us to hypothesize that palate shelf obstruction in these embryos might result from defects in tongue/mouth movement function *in utero*. Vertebrate embryos begin spontaneous neuromuscular movement of the mandible and tongue before birth (Walker, 1969; Wragg et al., 1972). In mouse, these movements begin at E14.5. To determine if retinoid-deficient embryos have a defect in spontaneous mandible/tongue movement, we evaluated embryo movement through *in utero* ultrasound. Whereas all other experiments in this study were performed on crosses that generated 50% control embryos and 50% mutant embryos in a litter, for ultrasound analysis of fetuses *in utero* a variant crossing strategy was used in order to have certainty regarding the genotype of embryos analyzed. With the ultrasound variant crossing strategy, the pre-tamoxifen genotype of mutant embryos (*Rdh10^flox/flox^*) differs from the pre-tamoxifen genotype of mutant embryos analyzed in other experiments (*Rdh10^delta/flox^*); however, once tamoxifen is administered and Cre excision inactivates the two *Rdh10^flox^* alleles, the genotype of mutant embryos generated by this cross becomes *Rdh10^delta/delta^*, identical to the post-tamoxifen genotype of mutant embryos analyzed in all other experiments of this study (Table S1). For analysis of control embryos *in utero*, litters of *Rdh10^+/+^* homozygous wild-type embryos were examined. In each case, tamoxifen was administered at E8.5, consistent with all previous experiments in this study.

**Fig. 4.**
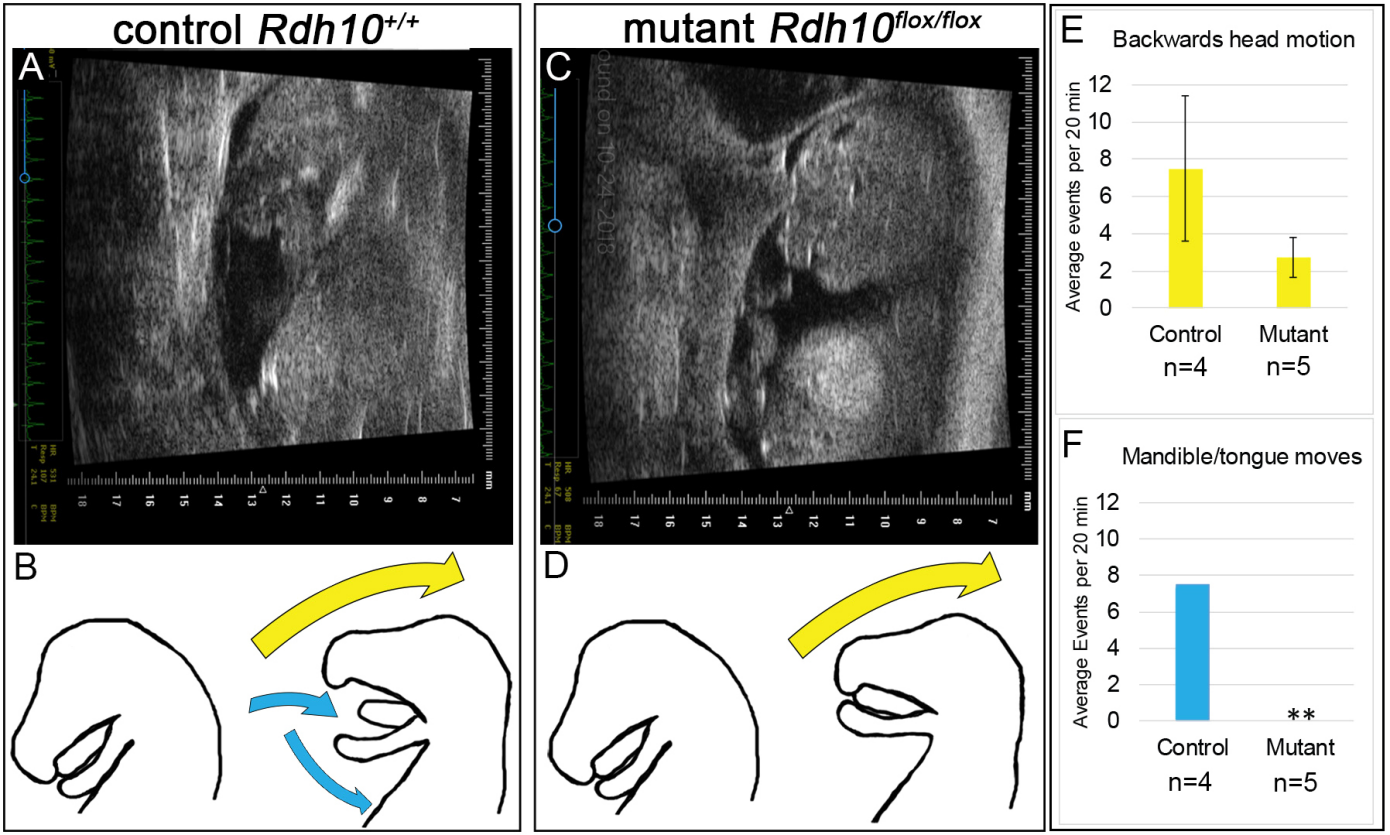
**Ultrasound analysis reveals that spontaneous mouth movement is restricted in *Rdh10^delta/flox^* mutant embryos.** Ultrasound was performed on E14.5 embryos *in utero* to evaluate spontaneous fetal mouth movement. Spontaneous movement of the head was detected in both control and mutant embryos, but mouth opening and tongue withdrawal was only observed in control embryos. (A) Still image from an ultrasound of an *Rdh10^+/+^* control embryo. (B) Schematic drawing depicts movement observed in control embryos. Each movement event in control embryos includes opening of the mandible and withdrawal of the tongue (blue arrows), with simultaneous backwards extension of the head (yellow arrow) (see Movie 1). (C) Still image from an ultrasound of an *Rdh10^flox/flox^* mutant embryo. (D) Schematic drawing depicts movement observed in *Rdh10^flox/flox^* mutant embryos. Mutant embryo movement is limited to backwards extension of the head (yellow arrow). Mandible opening and tongue withdrawal are not observed in mutant embryos (see Movie 2). (E) Both control and mutant embryos exhibited backwards head motion with an average frequency of 2.5–7 movements per 20 min observation interval. The frequency of head movement was not significantly different between control and mutant embryos. (F) Control embryos exhibited mouth opening and tongue withdrawal with each head movement (average frequency 7 openings per 20 min observation interval). No mouth opening or tongue withdrawal was observed in mutant embryos. The difference in frequency of mouth opening was significantly different between control and mutant embryos using Fisher's exact test for independence; **P≤0.01.

To evaluate fetal mouth movement for *Rdh10^+/+^* control and *Rdh10^flox/flox^* mutant embryos *in utero*, ultrasound analysis was performed at E14.5 (Fig. 4A,C). For each pregnant dam, a single embryo, oriented with a sagittal profile suitable for viewing, was analyzed for a 20 min period. For both control and mutant fetuses, periodic spontaneous movements were observed. In both groups, the head would jerk quickly back away from the abdominal cavity, a motion that created space for the mandible to open (Fig. 4B,D yellow arrows). In control embryos, each head movement was accompanied by opening of the mandible and a retraction of the tongue (Fig. 4B, blue arrows, see Movie 1). By contrast, in mutant embryos the backward head extension occurred, but was not accompanied by detectable mouth opening or tongue movement. Instead, the jaw remained closed and the tongue appeared inactive (Fig. 4D, see Movie 2).

In control embryos, backwards head extension with accompanying mandible/tongue movement was observed with an average frequency of 7 openings per 20 min observation interval (Fig. 4E,F). In mutant embryos, with head-only movement and immobile mandible/tongue, head-only movement events occurred at an average frequency of 2.5 events per 20 min observation interval (Fig. 4E,F). For one mutant embryo, spontaneous head movement was not detected, although the embryo did have a viable heartbeat. These data demonstrate that *Rdh10* and RA signaling are not essential for spontaneous fetal backward head extension, but are necessary for functional opening of the mandible and withdrawal of the tongue.

### *Rdh10^delta/flox^* mutant embryos have defects in motor nerves of the posterior pharyngeal arches

**Fig. 5.**
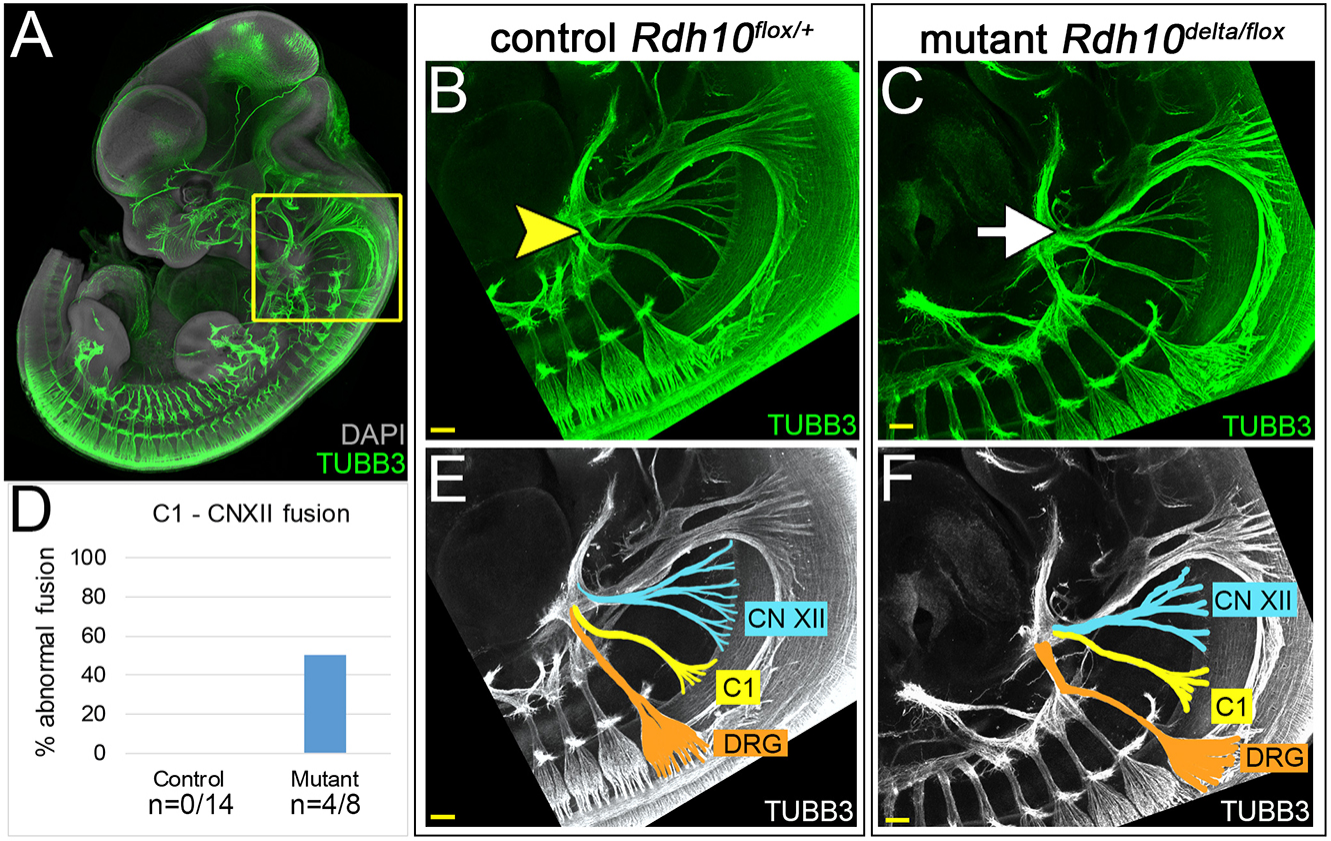
**Pharyngeal arch motor nerves are misrouted in *Rdh10^delta/flox^* mutant embryos.** (A) Wild-type E11.5 embryo immunostained whole mount for TUBB3 reveals all nerves. The yellow box defines the pharyngeal region shown in (B,C,E,F). (B) In *Rdh10^flox/+^* control embryos (B,E) motor nerve C1 routes posteriorly to plex with other cervical nerves behind the anterior-most dorsal root ganglion before turning superiorly towards the pharyngeal region (yellow arrowhead). (C,F) In *Rdh10^delta/flox^* mutant embryos the motor nerve C1 does not track posteriorly behind the anterior-most dorsal root ganglion, but is misrouted to fuse directly with CN XII (white arrow). (E,F) Color-coded images of control (E) and mutant (F) embryos to highlight the misrouting of C1 fusing with CN XII in mutant embryos. (D) The frequency of aberrant fusion of C1 to CN XII was 50% for mutant nerves (*n*=4/8). Aberrant fusion was never observed in nerves of control embryos (*n*=0/14). Scale bars: 100 µm.

We investigated the possibility that lack of fetal mouth/tongue movement in *Rdh10* mutant embryos was associated with the abnormal development of motor nerves serving the tongue and pharyngeal swallowing musculature. To assess motor nerve development in control and mutant embryos, we performed whole-mount immunostaining for tubulin β-3 chain (TUBB3) to identify neurons of E11.5 embryos (Fig. 5A). The motor nerve CN XII innervates the muscles of the tongue. The motor nerve C1 innervates pharyngeal muscles, including the geniohyoid and thyrohyoid, which work synergistically to move the hyoid bone and larynx during swallowing. CN XII outgrows from the ventral neural tube anterior to C1, and routes along a characteristic curving arc towards the tongue. C1 emerges from the neural tube posterior to CN XII and tracks along a more posterior route to join with the ventral rami of the cervical spinal nerves (C1-C4) before turning anteriorly to target the pharyngeal musculature. *Rdh10^flox/+^* control embryos had normal routing of the pharyngeal nerves. In these embryos, CN XII fibers followed an independent pathway curving anteriorly towards the tongue, whereas C1 fibers routed posteriorly before disappearing from view behind the first dorsal root ganglion (Fig. 5B,E). By contrast, *Rdh10^delta/flox^* mutant embryos exhibited dysmorphic routing of CN XII and C1, with the two motor nerves fusing abnormally (Fig. 5C,F). In such cases C1 did not travel posteriorly to join the cervical plexus, but instead fused directly with CN XII (Fig. 5C,F). This abnormal fusion of C1 and CN XII was observed in 50% of the mutant nerves (*n*=4/8), but was never observed in the nerves of control littermates (*n*=0/14) (Fig. 5D). The dysmorphic fusion of C1 to CN XII most likely reduces function of the tongue and pharyngeal swallowing muscles innervated by these nerves. These data suggest that abnormal motor nerve patterning could contribute to the defects in fetal tongue/mandible movement observed in *Rdh10^delta/flox^* mutant embryos.

### Retinoid-deficient embryos develop defects in the pharyngeal skeleton

**Fig. 6.**
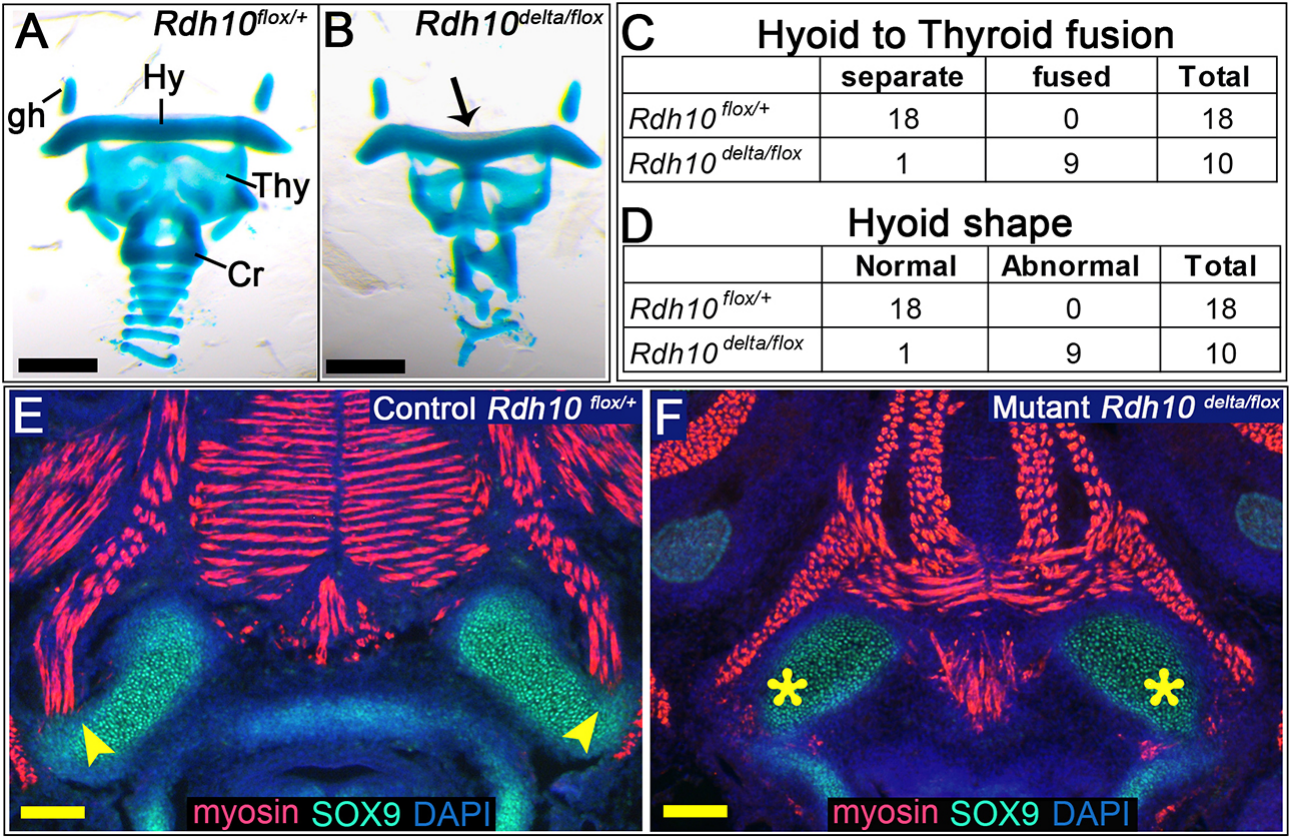
***Rdh10^delta/flox^* mutant embryos have defects in pharyngeal skeletal primordia.** Skeletal preparation of isolated pharyngeal cartilages from *Rdh10^flox/+^* control embryos (A) and *Rdh10^delta/flox^* mutant embryos (B) at E16.5. (B) In mutant embryos the laryngeal prominence of the thyroid cartilage was abnormally fused to the primordium of the hyoid bone (black arrow). (C) Abnormal fusion of the thyroid cartilage to hyoid primordium was observed in mutant embryos (*n*=9/10), but was never detected in control samples (*n*=0/18) (Fisher's exact test for independence *P*≤0.05). (B,D) In addition to the abnormal fusion, the hyoid primordia in mutant embryos also had an abnormal distinctive ‘M’ shape (black arrow, *n*=9/10) compared with the hyoid of control embryos, which did not exhibit the M shape (*n*=0/18) (Fisher's exact test for independence *P*≤0.05). (E,F) Transverse sections through tongues of E14.5 control (E) and mutant embryos (F) were immunostained with antibodies against myosin (muscle primordia) and SOX9 (cartilage primordia). (E) In control embryos, muscle fibers oriented towards and abutted the greater horn of the hyoid (yellow arrowheads). By contrast, in mutant embryos muscle fiber contact to the dysplastic greater horns of the hyoid was not evident (F, yellow asterisks). (A,B) Black scale bars: 500 µm. (E,F) Yellow scale bars: 100 µm. Cr, cricoid cartilage primordium; gh, greater horn of the hyoid bone primordium; Hy, hyoid bone primordium; Thy, thyroid cartilage primordium.

To further explore underlying defects that could contribute to lack of fetal mouth movement in *Rdh10* mutant embryos, we examined the morphology of the pharyngeal skeletal elements necessary for anchoring the tongue and swallowing muscles. These skeletal elements include the hyoid bone and the thyroid and cricoid cartilages. The primordia of the pharyngeal skeleton of E16.5 *Rdh10^delta/flox^* mutant and *Rdh10^flox/+^* control embryos was assessed by staining with Alcian Blue and Alizarin Red. Analysis of stained specimens revealed that *Rdh10^delta/flox^* mutant embryos had morphological defects in all elements of the pharyngeal skeleton (Fig. 6A,B). Defects observed in *Rdh10^delta/flox^* mutant embryos included ectopic fusion of the hyoid primordium to the laryngeal prominence of the thyroid cartilage (Fig. 6C) and a dysmorphic hyoid primordium with a shape of a gentle ‘M’ (Fig. 6D). The striking pharyngeal skeleton phenotypes observed in *Rdh10^delta/flox^* mutant embryos parallel those previously described for other models of retinoid deficiency (Luo et al., 1996; See et al., 2008; Vermot et al., 2003).

We next examined the attachment of the tongue muscles to the pharyngeal cartilages in *Rdh10^flox/+^* control and *Rdh10^delta/flox^* mutant embryos using paraffin sections and immunostaining. E14.5 embryos were sectioned transversely at the level of the hyoid bone and stained for myosin (to visualize muscle primordia) and for SOX9 (to visualize cartilage primordia). In control embryos, myosin-positive fibers were oriented in the direction of, and abutted, the greater horn of the hyoid, suggesting that these muscles attached appropriately to the bone primordium (Fig. 6E). By contrast, in *Rdh10^delta/flox^* mutant embryos, myosin-positive fibers did not appear to contact the greater horn of the hyoid (Fig. 6F). The lack of definitive muscle contact to the malformed hyoid primordium suggests that the muscle-anchoring attachment could be impaired in mutant embryos. In these transverse sections at the level of the hyoid bone, there is a detectable difference in orientation of tongue muscle fibers between mutant and control tongues, which corresponds to the abnormal arched positioning of the tongue in mutant embryos (Fig. S2). The observation that *Rdh10^delta/flox^* mutant embryos have dysplastic pharyngeal cartilages, with reduced or absent muscle contact, suggests that defects in patterning of the pharyngeal skeleton could contribute to the defects in fetal tongue/mandible movement observed in *Rdh10^delta/flox^* mutant embryos.

### Inactivation of *Rdh10* disrupts pharyngeal anterior-posterior patterning genes, consistent with other RA deficiency models

To gain understanding of the underlying gene expression changes contributing to the pharyngeal patterning abnormalities and loss of fetal mouth movement, we next assessed RA signaling and gene expression in pharyngeal tissues of *Rdh10^flox/+^* control and *Rdh10^delta/flox^* mutant embryos.

**Fig. 7.**
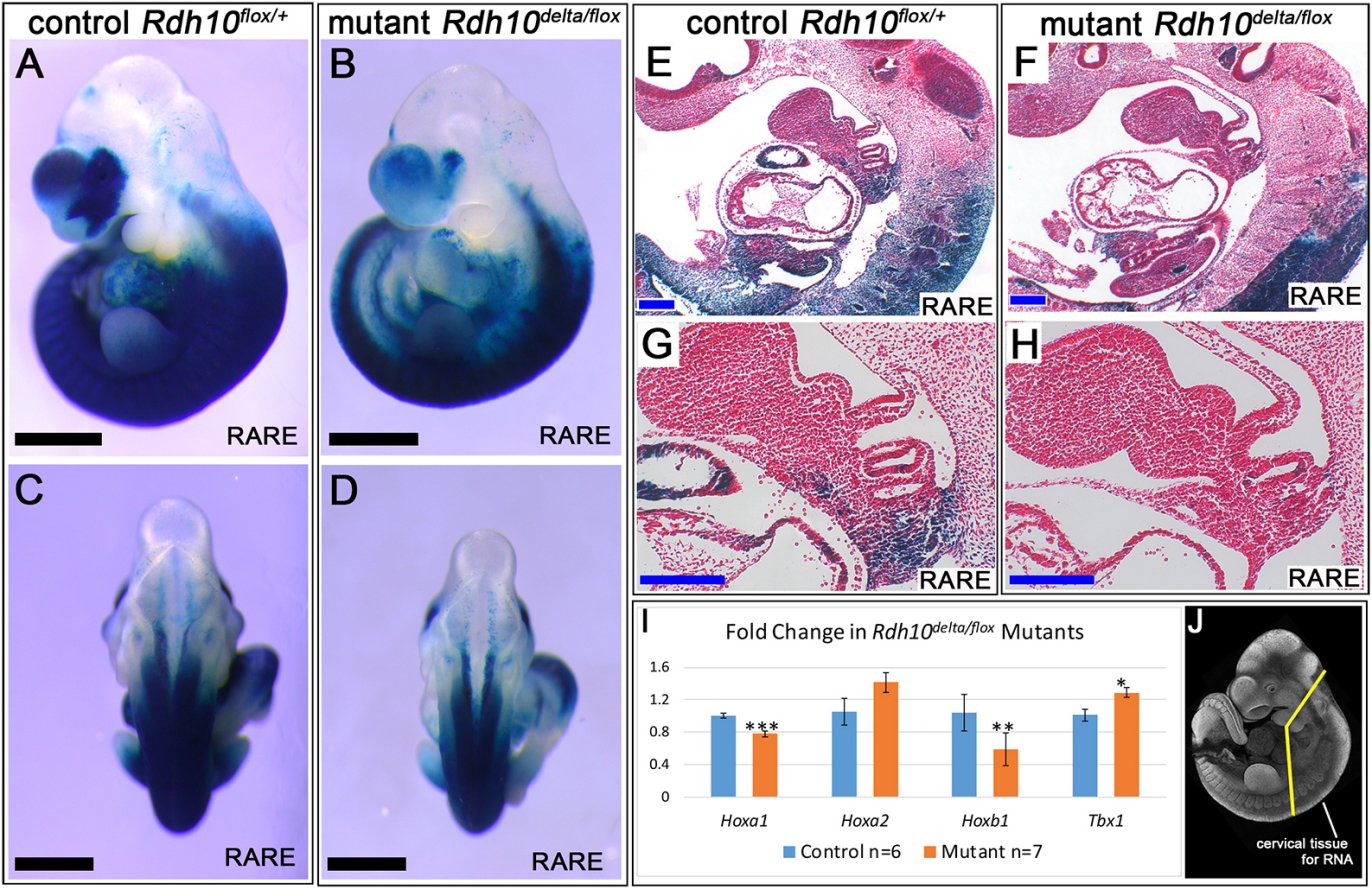
***Rdh10^delta/flox^* inactivation reduces RA signaling and modifies expression of *Hoxa1*, *Hoxb1* and *Tbx1* in pharyngeal tissues.** (A-H) E10.5 embryos carrying the RARE-*lacZ* reporter transgene were stained whole mount with X-gal to show the pattern of RA signaling. (A,C, *n*=3) *Rdh10^flox/+^* control embryo in (A) side and (C) dorsal view. (B,D, *n*=5) Diminished RA signaling in *Rdh10^delta/flox^* mutants is evident in (B) side view. (E-H) Whole-mount stained embryos were sectioned to view the distribution of RA signaling in pharyngeal arch tissues. In the control embryo, RA signaling is active in the somitic mesoderm and pharyngeal mesenchyme (E,G). By contrast, in *Rdh10^delta/flox^* mutant embryos, somitic mesoderm and pharyngeal mesenchyme are predominantly negative for RA signaling (F,H). (I) Expression of pharyngeal patterning genes was assessed in *Rdh10^delta/flox^* mutant embryos (*n*=7), relative to control embryos (*n*=6) by qPCR. (J) RNA was prepared from cervical tissue microdissected from E10.5 embryos. In cervical tissue of mutant embryos, *Hoxa1* and *Hoxb1* expression was reduced to 60% that of controls, whereas *Tbx1* was increased to 130% relative to the control (I). Gene expression differences: *P*≤0.01 for *Hoxa1*, *Hoxb1* and *Tbx1* and *P*≥0.05 for *Hoxa2*. *P≤0.05, **P≤0.01, ***P≤0.05, via Student's *t*-test. (A-D) Black scale bars: 1 mm. (E-H) Blue scale bars: 200 µm.

For the *Rdh10* conditional mutant system used in this study (administered tamoxifen at E8.5), we have previously quantified the overall whole-embryo level of RA signaling activity in mutant embryos to be 30% that of control littermates at E11.5 (Metzler et al., 2018). Here, we use the RARE-*lacZ* reporter transgene to assess the spatial distribution of RA signaling activity within the pharyngeal region at E10.5 (Fig. 7A-H). In whole-mount specimens, RARE-*lacZ* staining is visibly reduced in *Rdh10^delta/flox^* mutant embryos relative to *Rdh10^flox/+^* control littermates (Fig. 7B,D, *n*=5 mutant embryos). The reduction in RARE–*lacZ* staining is particularly evident within the ventral tissues of mutant embryos. Analysis of sagittal sections through the pharyngeal region reveals that tissues exhibiting reduced RA signaling in mutant embryos include somitic mesoderm and posterior pharyngeal arch mesenchyme (Fig. 7E-H).

RA is known to regulate expression of pharyngeal patterning genes (Bel-Vialar et al., 2002; Deschamps and van Nes, 2005; Diez del Corral and Storey, 2004; Gavalas et al., 1998; White et al., 2000; White et al., 1998). To understand the etiology of the pharyngeal abnormalities in retinoid-deficient embryos, we examined the expression of key pharyngeal patterning genes in control and mutant embryos. To this end, we performed quantitative real-time PCR (qPCR) for *Hoxa1*, *Hoxa2*, *Hoxb1* and *Tbx1* on isolated cervical tissues that included the second to sixth arches of E10.5 embryos (Fig. 7J). *Hoxa1* and *Hoxb1*, both of which are directly regulated by RA signaling, were significantly downregulated in *Rdh10^delta/flox^* mutant embryos to 60% that of controls (Fig. 7I). Conversely, expression of *Tbx1*, a transcription factor known to be negatively regulated by RA signaling, was increased in *Rdh10^delta/flox^* mutant pharyngeal tissues to 130% the level of controls (Fig. 7I). Expression of *Hoxa2*, which is known to be important for developmental patterning of pharyngeal musculature, was not significantly different between mutant and control specimens (Fig. 7I). We also examined expression of *Hoxa3*, a gene required for the development of pharyngeal cartilages (Chojnowski et al., 2016; Mulder et al., 1998); however, no significant difference was observed (data not shown). These data demonstrate that RA signaling is reduced in pharyngeal tissues and expression of key pharyngeal patterning genes is altered in mutant embryos. These changes in RA signaling and gene expression provide an underlying molecular mechanism for the abnormal development of the pharyngeal skeleton and motor nerves observed in *Rdh10^delta/flox^* mutant embryos.

## DISCUSSION

Fetal mouth movement has long been postulated to have a role in palate development (His, 1901). In mice, fetal mouth and tongue movements have been observed directly, and are noted to begin at the same gestational stage as palate shelf elevation (Walker, 1969). Defects in musculoskeletal anchoring of the tongue that would impair tongue function are correlated with cleft palate in *Hoxa2* mutant mice (Barrow and Capecchi, 1999). Cleft palate is also observed in mice with defects in neurotransmission owing to mutation of *Gad1*, *Gad2*, *Gabrb3* or *Slc32a1*, which disrupt production, transport and receptor signaling by the inhibitory neurotransmitter γ-aminobutyric acid (Asada et al., 1997; Condie et al., 1997; Culiat et al., 1995; Homanics et al., 1997; Oh et al., 2010; Wojcik et al., 2006). Although these studies support the idea that defects in fetal mouth movement can contribute to cleft palate, to date, direct analysis of fetal mouth immobility in conjunction with cleft palate has been reported in only one experimental study disrupting neurotransmission in mice (Tsunekawa et al., 2005), and in a case study of a human patient with syngnathia (Laster et al., 2001). Here, by directly demonstrating restricted fetal mouth movement in conjunction with palate shelf obstruction in retinoid-deficient embryos, we extend the range of defects that can be shown to impair fetal mouth function. By demonstrating that fetal mouth movement can be inhibited by defects in the morphogenesis of pharyngeal skeletal elements and routing of pharyngeal motor nerves, we have expanded understanding of the range of etiological mechanisms that can disrupt the fetal mouth activity necessary for palate shelf elevation.

**Fig. 8.**
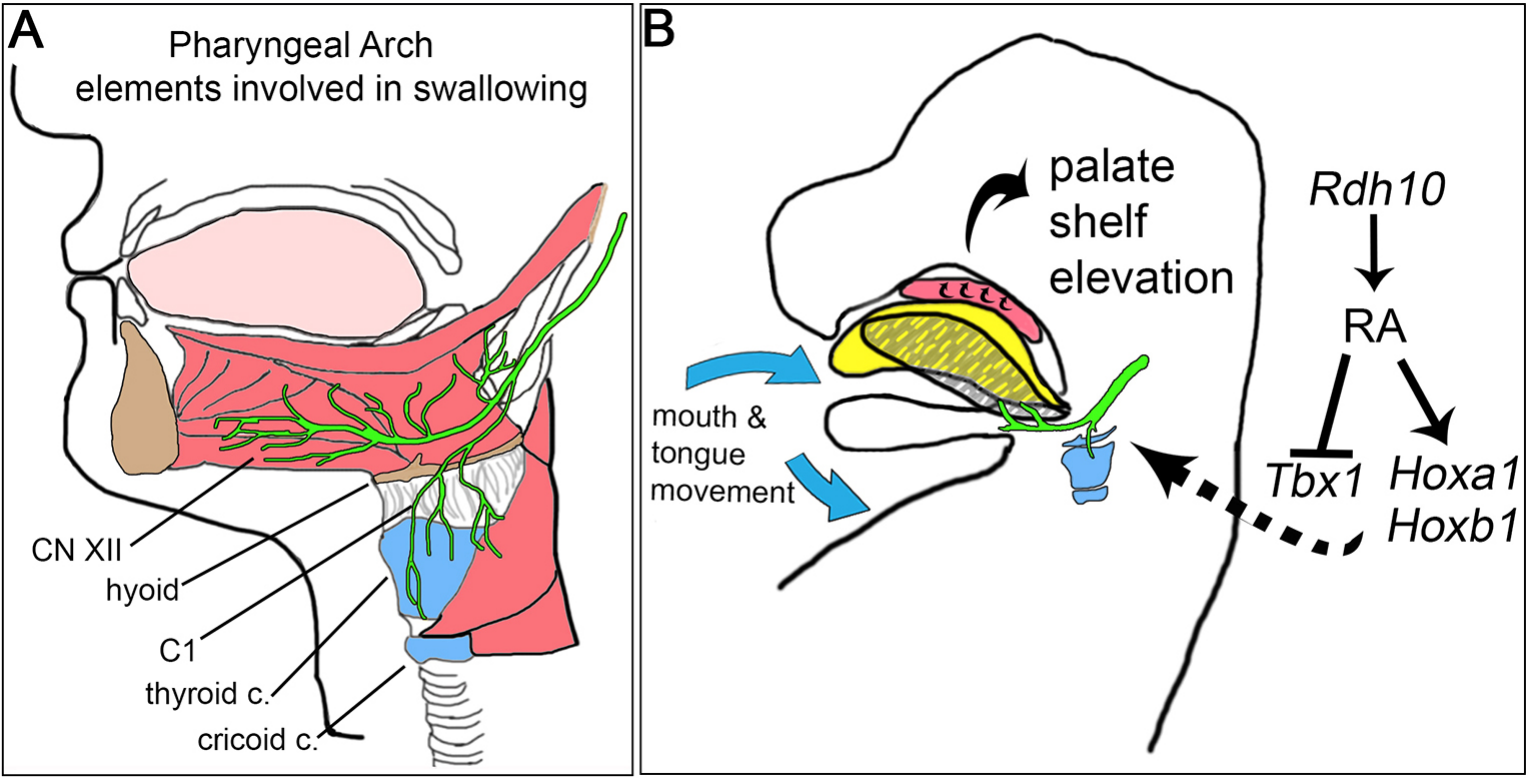
**The development of pharyngeal nerves and skeletal elements crucial for fetal mouth movement and palate formation is dependent upon RDH10-mediated retinol metabolism and RA signaling.** (A) Pharyngeal arch derivatives involved in mouth movement and swallowing include the hyoid bone, the thyroid and cricoid cartilages, CN XII and C1, and muscles that attach the tongue and mandible to the pharyngeal skeleton. (B) During embryogenesis, RA signaling, which depends on *Rdh10-*mediated retinol metabolism, is essential for the proper regulation of pharyngeal patterning genes, including *Tbx1*, *Hoxa1* and *Hoxb1.* These genes are crucial for patterning the anterior-posterior axis during embryonic development. The patterning of the pharyngeal region allows for proper development of the motor nerves, cartilage and muscle attachments that enable spontaneous fetal mouth movement. This movement allows the resting tongue (yellow) to depress and retract (hatched gray). The retraction moves the tongue out of the way of the palate shelves, giving them room to elevate and fuse to close the dome of the oral cavity.

Our results link RA signaling to palatogenesis through regulation of anterior–posterior patterning, which is crucial for formation of the neurostructural anatomy of the pharyngeal region (Fig. 8A,B). The study highlights that palate development depends on proper morphogenesis and function of peripheral motor nerves, CN XII and C1, along with the pharyngeal skeleton, including the hyoid bone, the thyroid cartilage and cricoid cartilage, which work synergistically to move and anchor the muscles of the tongue and mandible (Fig. 8A). It is known that CN XII establishes connection to the tongue and initiates the onset of motor nerve activity, mouth opening and tongue withdrawal at the time of palate elevation (Walker, 1969; Wragg et al., 1972). Here, we link the RDH10–mediated metabolism of vitamin A, together with RA regulation of anterior–posterior patterning genes, to these important developmental events (Fig. 8B).

Tongue interference with palate shelf closure has been implicated in cleft palate independent of movement defects. Increased tongue height has been suggested to have a role in inhibiting the palate shelves from elevating (Song et al., 2013). The microCT analysis of retinoid-deficient embryos presented here sheds new light on the 3D morphology of the tongue in the context of obstructing palate shelf elevation. The tongues of *Rdh10* mutant embryos are slightly smaller by volume, but have a heightened posterior aspect. The 3D rendering reveals that a heightened tongue appearance could be the result of posterior tongue contraction caused by a defect in neurostructural ability of the embryo to depress the tongue. These observations suggest that interpretation of tongue height from 2D histological sections should be made with caution.

Previous studies have shown that defects in neurotransmission are strongly correlated with cleft palate (Asada et al., 1997; Condie et al., 1997; Culiat et al., 1995; Homanics et al., 1997; Oh et al., 2010; Wojcik et al., 2006) and that such defects can be attributed to a lack of spontaneous fetal mouth movement (Tsunekawa et al., 2005). Here, we show that defects in mouth movement can result from mispatterning of peripheral motor nerves rather than as a loss of neurotransmitter function. Moreover, the movement defect we describe here is isolated to the mandible and tongue, whereas backwards head extension remained active. The presence or absence of head movement in previous neurotransmitter studies was not specified.

In human cleft palate patients and mouse models, pharyngeal defects have been observed in association with cleft palate. Abnormalities in the location and formation of the hyoid bone have been observed in cleft palate populations (Rajion et al., 2006; Wahaj et al., 2014). Phenotypes associated with 22q11.2 deletion syndrome in human patients include pharyngeal abnormalities, postnatal dysphagia and cleft palate (LaMantia et al., 2016; McDonald-McGinn et al., 2015; Scambler, 2010). In a mouse model of this syndrome, pharyngeal nerve-patterning defects have been noted (Karpinski et al., 2014). Analysis of mouse mutant phenotypes has revealed that postnatal difficulty in swallowing can be attributed to loss of function of CN XII (Karpinski et al., 2014; Wang et al., 2017). We hypothesize that, in some cases, abnormalities in morphogenesis of the pharyngeal skeleton, or neurological defects that cause postnatal dysphagia, might also contribute to the formation of cleft palate by disrupting fetal mouth and tongue activity *in utero*.

One important gene disrupted in 22q11.2 deletion syndrome is *TBX1*, which is inversely regulated by RA signaling (Merscher et al., 2001; Roberts et al., 2005; Scambler, 2010; Yutzey, 2010). A link between 22q11.2 deletion syndrome and perturbation of RA signaling has been well established (Yutzey, 2010). Mouse models with disrupted RA signaling have phenotypes reminiscent of 22q11.2 deletion syndrome (Niederreither et al., 2003; Vermot et al., 2003). Excess RA downregulates *Tbx1* expression, whereas reduced RA results in overexpression of *Tbx1* (Roberts et al., 2005; Ryckebusch et al., 2010). Because pharyngeal arch development depends on a precise balance between *Tbx1* and RA, we suspect that the cause of cleft palate in 22q11.2 deletion syndrome might be related to pharyngeal patterning defects similar to those we observe in the retinoid-deficient *Rdh10^flox/delta^* mutant embryos, which have excess *Tbx1*. Perhaps correlation of cleft palate and pharyngeal arch defects in other models like 22q11.2 deletion syndrome can be understood through evaluating the presence of spontaneous mouth movement *in utero*.

By expanding the range of anomalies with demonstrable fetal mouth movement defects and cleft palate in mice, this study suggests that the range of possible defects contributing to cleft palate etiology in humans should likewise be extended. Although there are some structural differences between the developing oral/palatal regions of mouse fetuses relative to human fetuses (Yu et al., 2017), it seems plausible that insufficient fetal tongue movement owing to defects in pharyngeal development could obstruct palate shelf elevation in humans, as it does in mice. Further investigation of this mechanism is important as it could have ramifications for enabling early detection and preventing birth defects. More attention must be given to the optimization of RA levels during pregnancy, as counseling objectives might be improved through modulation of this dynamic and sensitive signaling pathway.

## MATERIALS AND METHODS

### Mouse strains

*Mus musculus* laboratory mice were used for all experiments in this study. The *Rdh10* mutant strains used in this study have been previously described (Sandell et al., 2012b). All mutant *Rdh10* alleles were derived from *Rdh10^Bgeo/+^* embryonic stem cells obtained from the trans-NIH Knockout Mouse Project (KOMP) Repository, a National Center for Research Resources - National Institutes of Health (NCRR-NIH)-supported strain repository (www.komp.org; email service@komp.org). For the experiments described in this study, the *Rdh10^flox/flox^* and *Rdh10^delta/+^* mice were bred extensively to FVB/NJ such that their background is mixed with a significant contribution of FVB/NJ. Additional mouse strains used were FVB/NJ, RARE-*lacZ* and Cre-ERT2, all obtained from Jackson Laboratories and maintained at the University of Louisville. Genotyping of all *Rdh10* alleles and transgenes, from tissue samples of embryos and adult breeder animals, was performed by the commercial genotyping service Transnetyx. The mutant and transgenic mouse strains used in this study are as follows: *Rdh10^flox/flox^* and *Rdh10^delta/+^* (both mixed background, primarily FVB/NJ) (Sandell et al., 2012b), Cre-ERT2 *Gt(ROSA)26Sor^tm1(cre/ERT2)Tyj^* (mixed background) (Ventura et al., 2007), and RARE-*lacZ* Tg(RARE-Hspa1b/*lacZ*)12Jrt (mixed background) (Rossant et al., 1991).

All animal experiments described in this study were reviewed by the Institutional Animal Care and Use Committee at the University of Louisville and were performed according to the approved protocol.

### Genetic crosses and conditional *Rdh10* inactivation by tamoxifen administration

For timed matings to obtain embryos, the day of the vaginal plug is considered E0.5. For all experiments in this study, with the exception of the ultrasound analysis, the following genetic cross was used in timed matings to produce control and mutant embryos within the same litter. Homozygous *Rdh10^flox/flox^*; Cre–ERT2/Cre-ERT2 mice were paired with *Rdh10^delta/+^* mice. Litters produced by such crosses have 50% of embryos with a pre-tamoxifen genotype of *Rdh10^flox/+^* ‘control’, which are heterozygous for the haplosufficient wild-type allele of *Rdh10*. The remaining 50% embryos from such a cross have a pre-tamoxifen genotype *Rdh10^delta/flox^* ‘mutant’, which are heterozygous for a deleted allele and a conditional floxed allele of *Rdh10*. All embryos have a single copy of Cre-ERT2. For all time-mated animals, a single dose of tamoxifen was administered at E8.5 via maternal oral gavage to activate Cre recombinase and delete *Rdh10* exon 2 from *Rdh10^flox^* alleles. Because Cre-ERT2 excision of *Rdh10* exon 2 converts the *Rdh10^flox^* allele into the *Rdh10^delta^* allele in all tissues of the embryo, control embryos that are *Rdh10^flox/+^* pre-tamoxifen are converted to heterozygous *Rdh10^delta/+^* post-tamoxifen, and mutant embryos that are *Rdh10^flox/delta^* pre-tamoxifen are converted to *Rdh10^delta/delta^* genotype post-tamoxifen (Table S1). Throughout the text and figures, we refer to the genotype using the ‘pre-tamoxifen’ state.

For the ultrasound experiment, a variation of the cross described above was performed so as to generate litters of embryos with a consistent genotype throughout the litter. For the ultrasound experiments, *Rdh10^flox/flox^* Cre-ERT2/Cre-ERT2 were mated with *Rdh10^flox/flox^* to generate litters in which all embryos were *Rdh10^flox/flox^* with a single copy of Cre-ERT2. Although the pre-tamoxifen genotype of these ‘mutant’ embryos differs from that of other experiments of the study, the post-tamoxifen genotype of these mutant embryos is identical to that of mutant embryos in all other experiments of this study (Table S1). For control embryos for the ultrasound experiment, *Rdh10^+/+^* were mated with *Rdh10^+/+^* to generate litters of all control embryos with the genotype *Rdh10^+/+^*. For the ultrasound experiment control embryos were homozygous *Rdh10^+/+^*, whereas control embryos of other experiments were heterozygous *Rdh10^flox/+^* that have no detectable cleft palate phenotype.

Each pregnant dam was administered an oral gavage dose of 5 mg of tamoxifen plus 2 mg progesterone in 250 µl of corn oil. Tamoxifen was prepared by first dissolving 20 mg/ml of tamoxifen (Sigma-Aldrich, #T5648) in absolute ethanol to a concentration of 500 µg/µl and subsequently diluting to a final concentration of 20 mg/ml in corn oil (VWR, #700000-136). The solution was vortexed every 30 min and incubated at 55°C until crystals were dissolved (∼3-4 h). Once tamoxifen was dissolved, progesterone (Sigma Aldrich, #P3972) was added to a final concentration of 8 mg/ml and the mixture incubated at 55°C (with vortexing every 15 min) until the progesterone dissolved (∼30 min). Aliquots of the solution were then placed into single-dose tubes and stored at −20°C for up to 2 weeks.

### Nuclear fluorescence imaging of palate tissues

Morphology of whole-mount palates and maxillary explants were imaged by nuclear fluorescence staining and fluorescence stereomicroscopy (Sandell et al., 2012a). Whole-mount specimens were fixed in 4% paraformaldehyde overnight, rinsed in PBS and stained in DAPI dilactate at a final working concentration of 10 nM in PBS solution at room temperature, with gentle rocking overnight. DAPI-stained specimens were imaged using a UV light source on a Leica stereomicroscope.

### Histology by H&E staining

For paraffin sectioning and histology, embryos were harvested and fixed overnight in 4% paraformaldehyde followed by dehydration through a series of increasingly concentrated ethanol solutions: 25%, 50%, 70% and 100%. Embryos were processed into paraffin, embedded and sectioned. Before staining, slides with embryo sections were deparaffinized and baked for 30 min at 58°C, followed by rehydration through xylene and ethanol into PBS. For H&E staining, samples were immersed in hematoxylin stain (VWR, #15204-152) for 8 min. Sections were placed under running tap water for 5 min and then placed into acid alcohol (VWR, #15204-234) for 30 s. Slides were rinsed again under running tap water for 2 min, transferred to lithium carbonate for 45 s and finally washed with tap water for 5 min. Sections were dipped in 80% ethanol 10 times, then placed in eosin for 40 s followed by dehydration back through an ethanol series into xylene. Finally, Permount Mounting Medium (VWR, #100496-550) was applied and specimens were covered with coverslips.

### Immunostaining of paraffin-sectioned and whole-mount embryos

Immunostaining of paraffin and whole-mount tissues was carried out as described previously (Abashev et al., 2017). For sections, antigen retrieval was performed and specimens were blocked in 5% lamb serum for 2 h. Specimens were stained with primary antibodies overnight, washed and stained with secondary antibodies for 1 h. After washing off secondary antibodies, slides were stained for 10 min with DAPI. Stained slides were mounted in Prolong Gold (Thermo Fisher, P36930) and covered with coverslips. For whole-mount immunostaining, embryos were first permeabilized for 2 h in Dent's bleach (methanol: 30% H_2_O_2_: DMSO, 4:1:1). After permeablization, embryos were rehydrated through a graded series of methanol solutions into PBS. Samples were blocked in 0.1 M Tris pH7.5, 0.15 M NaCl with blocking reagent (Perkin Elmer, FP1020). Primary antibody hybridization was performed in blocking solution overnight at 4°C with rocking. The next day, unbound primary antibody was removed with a series of 5×1 h washes in PBS at room temperature. Specimens were then incubated overnight in fluorescently conjugated secondary antibody in blocking solution at 4°C with rocking. Unbound secondary antibody was removed by 3×20 min washes in PBS, followed by 4 h nuclear staining with DAPI. Whole-mount specimens were then post-fixed in 4% paraformaldehyde for 20 min. Stained embryos were dehydrated through a graded series of methanol solutions into 100% methanol. Tissues were cleared by placing specimens in BABB (benzyl alcohol: benzyl benzoate, 1:2). Fluorescently immunostained embryos were then imaged by confocal microscopy on a Leica SP8 confocal microscope.

The primary antibodies used were anti-SOX9 (Abcam, #185966) 1:200, anti-myosin (DSHB, #BF-G6) 1:5 and neuronal class III β-tubulin monoclonal antibody (BioLegend, #801201) 1:1000. Secondary antibodies were fluorescently conjugated Alexa Fluor 660 (Invitrogen) and Alexa Fluor 546 (Invitrogen), each used at 1:300.

### *Ex vivo* culture of maxillary explants

Maxillary explant specimens from E13.5 embryos were microdissected free of mandible, tongue and brain tissues. One to three explants were placed in a glass scintillation vial with 6 ml of BGJb culture medium (Thermo Fisher, #12591038) supplemented with 2.8 mg/ml glutamine, 6 mg/ml BSA and 1% penicillin and streptomycin. No serum was added to the medium. The scintillation vial was flushed with a gas mixture (50% O_2_, 45% N_2_ and 5% CO_2_) and sealed using a silicone plug. The plugged vials were incubated on a Wheaton mini bench-top roller bottle system at a speed of 25 rpm in a humidified 37°C incubator for 3 days. Each day, vials were reflushed with the gas mixture for 2 min. At the end of the 3 day culture period, explants were fixed in 4% paraformaldehyde. Following fixation, specimens were whole-mount stained with DAPI and imaged by fluorescence microscopy (Sandell et al., 2012a).

### MicroCT analysis of embryos

Whole E14.5 embryos were fixed in 4% paraformaldehyde overnight. Embryos were then equilibrated in 50% ethanol overnight, transferred to 70% ethanol for 2.5 h and stained in phosphotungstic acid (PTA) stain for 8 days. The PTA stain solution is a heavy X-ray dense molecule that produces high contrast for soft tissues in X-ray analysis. After PTA staining, samples were placed in 70% ethanol for 2 h and then transferred to 100% ethanol for scanning. To prepare the PTA solution, 1 g of PTA dry powder (VWR, #AA40116-22) was dissolved in 100 ml of distilled water to make a 1% PTA stock solution. A 30 ml aliquot of this stock solution was then dissolved in 70 ml 100% ethanol to make a 0.3% PTA in 70% ethanol working stain solution.

Embryos were mounted inside a 1000 µl tapered pipette tip, sealed with paraffin wax at the bottom and filled with 100% ethanol. The embryo was gently wedged by gravity into the tapered end of the pipette. The pipette was then filled to the top with 100% ethanol and sealed with a wax film. Another pipette tip was cut in half and wedged into sculptor's clay on the microCT platform to serve as a holder. The embryo in the pipette tip was placed in the holder on the rotating platform of a Bruker microCT SkyScan 1174v2 compact X-ray microCT scanner. The settings used for scanning were as follows: pixel size 6.84 µm, rotation of 0.3° with an average of three frames, 50 kV and 800 µA. No filter was used. Bruker CT analysis software was used to visualize 2D sections from the microCT scan data files. Additionally, Imaris software was used for volume-rendering of the microCT data sets of control and mutant tongues.

### RARE-*lacZ* reporter gene staining with X-gal

To evaluate the tissue distribution of RA signaling, embryos were stained for β–galactosidase activity as whole-mount specimens, imaged and then processed and embedded in paraffin for sectioning. E10.5 RARE-*lacZ* reporter mouse embryos were harvested into ice cold PBS and fixed whole mount in 2% formalin plus 0.2% glutaraldehyde for 75 min on ice. After fixation, embryos were rinsed with β-galactosidase tissue rinse solution A (Millipore, #BG-6-G), then washed in solution A for 30 min at room temperature. Embryos were next rinsed with β-galactosidase tissue rinse solution B (Millipore, #BG-7-G) and washed in solution B for 5 min at room temperature. Fixed embryos were then drained and placed in stain solution: β-galactosidase tissue stain base solution (Millipore, #BG-8C) plus 1 mg/ml X-gal (Sigma-Aldrich, #B4252-250MG). Embryos were stained overnight at room temperature protected from light, then post-fixed in 4% paraformaldehyde overnight. Sections were counterstained with Nuclear Fast Red (VWR, #JTS635-1). After whole-mount imaging, embryos were processed into paraffin and sectioned.

### qPCR

Gene expression levels were quantified by qPCR. Cervical tissues including the posterior pharyngeal arches (second to sixth) were removed from E10.5 embryos by microdissection (Fig. 7J). Tissues were homogenized in RLT lysis buffer (Qiagen, #79216) with a syringe and needle. RNA was extracted from the tissue using RNeasy Mini kit (Qiagen, #74104) and further converted to cDNA using SuperScript III First-Strand Synthesis System (Invitrogen, #18080-051) and random hexamer primers. The following gene-specific qPCR primers were used for amplification of RNA: *Gapdh* (F) 5′-ACAGTCCATGCCATCACTGCC-3′, (R) 5′-GCCTGCTTCACCACCTTCTTG-3′; *Hoxa1* (F) 5′-CCCAGACGGCTACTTACCAGA-3′, (R) 5′-CATAAGGCGCACTGAAGTTCT-3′; *Hoxa2* (F) 5′-CTGAGTGCCTGACATCTTTTCC-3′, (R) 5′-GTGTGAAAGCGTCGAGGTCTT-3′; *Hoxb1* (F) 5′-GCCCCAACCTCTTTTCCCC-3′, (R) 5′-GACAGGATACCCCGAGTTTTG-3′; *Tbx1* (F) 5′-CTGTGGGACGAGTTCAATCAG-3′, (R) 5′-TTGTCATCTACGGGCACAAAG-3′. All primers used were validated to have an efficiency between 90% and 110%. The *Gapdh* gene was used for normalization of gene expression. Data were evaluated by the 2^−ΔΔCT^ method (Livak and Schmittgen, 2001). Significance was evaluated by the two-tailed Student's *t*-test assuming equal variance.

### Skeletal staining with Alizarin Red and Alcian Blue

For skeletal staining of bone and cartilage, embryos were harvested at E16.5. Embryos were decapitated and skin and organs were left intact. Embryos were rinsed in PBS and placed in ice-cold 95% ethanol for 1 h. Specimens were then transferred to fresh ethanol and rocked overnight at room temperature. Specimens were double-stained for cartilage and bone using Alcian Blue and Alizarin Red. The stain mixture was prepared in two separate stock solutions that were combined into a working mixture prior to staining. The stock solution of 0.4% Alcian Blue in 70% ethanol was prepared by first adding 0.4 g Alcian Blue (VWR, #200063-912) to 10 ml of 50% ethanol and placing in a 37°C water bath with occasional swirling until dissolved; once the Alcian Blue had dissolved, 25 ml of water and 65 ml of 95% ethanol were added. The stock solution of 0.5% Alizarin Red S in water was prepared by adding 0.5 g of Alizarin Red S (VWR, #97062-616) to 100 ml of water and swirling until dissolved. The combined working stain solution was then prepared from the two separate stock solutions. For 100 ml of working stain solution, 5 ml of 0.4% Alcian Blue in 70% ethanol was combined with 5 ml of glacial acetic acid (VWR, #BDH3098-3.8L), 70 ml of 95% ethanol, 20 ml of water and 1 ml of 0.5% Alizarin Red stock solution. Specimens were placed in the working stain solution for up to 8 days. At the completion of the staining period, specimens were rinsed in water. The non-bone and cartilage tissues were cleared by incubating in a series of potassium hydroxide (VWR, #BDH9262-500G) and glycerol (VWR, #AAAA16205-AP) solutions as follows: 4 h in 2% KOH then 30 min in 0.25% KOH, overnight in 20% glycerol/0.25% KOH, overnight in 33% glycerol/0.25% KOH and, finally, overnight in 50% glycerol/0.25% KOH. Once specimens were cleared to reveal bone and cartilage, mandibles were carefully dissected away from the skull. Specimens were then imaged on a Leica stereomicroscope and measured with Leica imaging software.

### Ultrasound imaging

Using a 2% concentration of isoflurane anesthesia, a pregnant mouse at E14.5 was placed in the supine position, a rectal temperature probe was placed, fur was removed and pre-warmed ultrasound gel was placed on the dam's abdomen. Once the dam's heart rate reached 500 bpm and the body temperature was 37°C, a 10 min window was observed to allow equilibration to the isoflurane. Using the VisualSonics 770 ultrasound system, an RMV 707B probe was placed on the ultrasound gel covering the dam's abdomen. A sagittal-positioned embryo was located and monitored for a 20 min period, during which the dam's body temperature was monitored and maintained. During this time, any movement of the embryo, namely mouth opening or backward extension of the head and neck, was documented. For each dam, only one embryo was monitored.

## Supplementary Material

Supplementary information
