# Supplementary material for: RDH10 function is necessary for spontaneous fetal mouth movement that facilitates palate shelf elevation
Source: Dis Model Mech. 2019 Jul 3;12(7):dmm039073. doi: 10.1242/dmm.039073 (PMC6679383; doi:10.1242/dmm.039073)
Supplement: Supplementary information [file dmm-12-039073-s1.pdf]

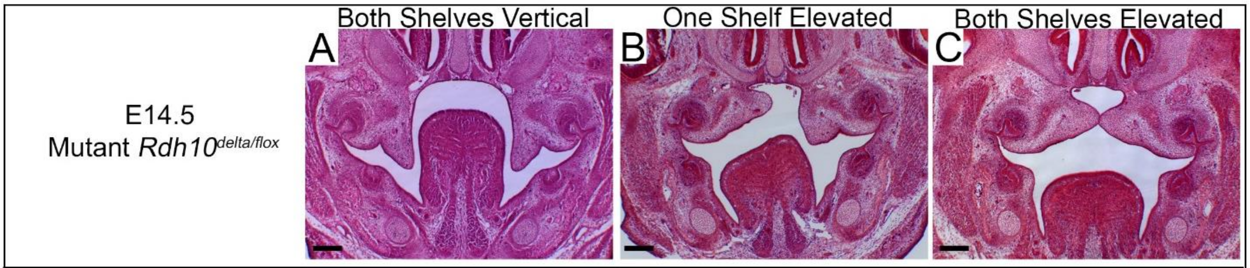

D

	Both Shelves Vertical	One Shelf Elevated	Both Shelves Elevated
Control *Rdh10* flox/+ (n=12)	8%	8%	84%
Mutant *Rdh10* delta/flox (n=15)	60%	6%	33%

Fig. S1. Frequencies of bilateral and unilateral palate shelf elevation in *Rdh10*$^{delta/flox}$ mutant and *Rdh10*$^{flox/+}$ control embryos at E14.5.

At E14.5, the stage when palate shelf re-orientation to the horizontal position occurs, control and mutant embryos had either both shelves oriented in the vertical position (A), one shelf elevated (B), or both shelves elevated (C). 82% of *Rdh10*$^{flox/+}$ control embryos had both palate shelves oriented horizontally, while only of 33% mutant embryos had both shelves in the horizontal orientation (D). Unilateral palate shelf elevation was observed at frequency of 8% in the control and 6% in the mutant (D). 60% of the mutants had shelves oriented in the horizontal position while only 8% of controls at this stage exhibited vertically oriented shelves (D). *Rdh10*$^{flox/+}$ control (n=12). *Rdh10*$^{delta/flox}$ mutant (n=15).

Disease Models & Mechanisms • Supplementary information

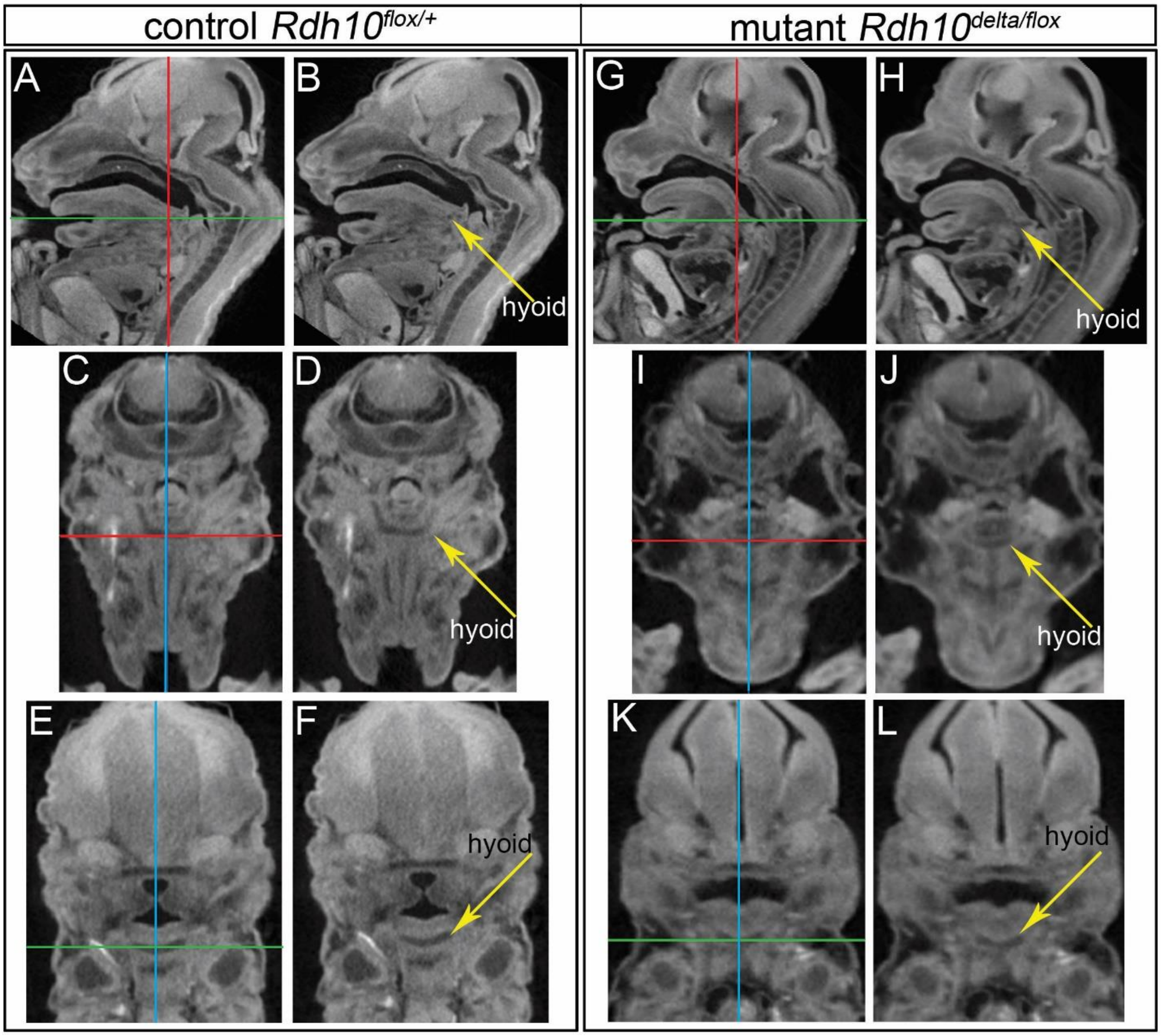

Fig. S2. Sagittal, transverse, and coronal views of μCT datasets highlight differences in tongue morphology and orientation relative to hyoid bone between *Rdh10*$^{flox/+}$ control and *Rdh10*$^{delta/flox}$ mutant embryos

Control and *Rdh10*$^{delta/flox}$ mutants have different morphology of craniofacial structures. A transverse section through control embryo at the level of the hyoid will look different than a corresponding section through a *Rdh10*$^{delta/flox}$ mutant at the same level. (A-B) Sagittal sections of control embryo at the midline corresponding to the blue line in (C, E). (C-D) Transverse sections of control at the level of the hyoid primordium, corresponding to the green line in (A, E). (E, F) Coronal sections of control embryo at the level of the hyoid primordium, corresponding to the red line in (A, C). (G-H) Sagittal sections of *Rdh10*$^{delta/flox}$ mutant embryos at the midline, corresponding to the blue line

Disease Models & Mechanisms • Supplementary information

in (I, K). (I, J) Transverse sections through *Rdh10*$^{delta/flox}$ mutant embryos at the level of the hyoid primordium, corresponding to the green line in (G, K). (K, L) Coronal sections through *Rdh10*$^{delta/flox}$ mutant embryos at the level of the hyoid primordium, corresponding to the red line in (G, I). All sections within each group (control or mutant) are retrieved from the whole dataset and generated from a single 3D location shown at the cross-hair to display three orthogonal views. Yellow arrow indicates position of hyoid primordia.

Table S1. Genotype of control and mutant embryos before and after administration of tamoxifen to activate Cre ERT2 excision.

	**Pre-Tamoxifen Genotype**	**Post-Tamoxifen Genotype**	**Experimental use**
control	*Rdh10*$^{+/+}$	*Rdh10*$^{+/+}$	Ultrasound
control	*Rdh10*$^{flox/+}$	*Rdh10*$^{delta/+}$	All but ultrasound
mutant	*Rdh10*$^{flox/delta}$	*Rdh10*$^{delta/delta}$	All but ultrasound
mutant	*Rdh10*$^{flox/flox}$	*Rdh10*$^{delta/delta}$	Ultrasound

Disease Models & Mechanisms • Supplementary information

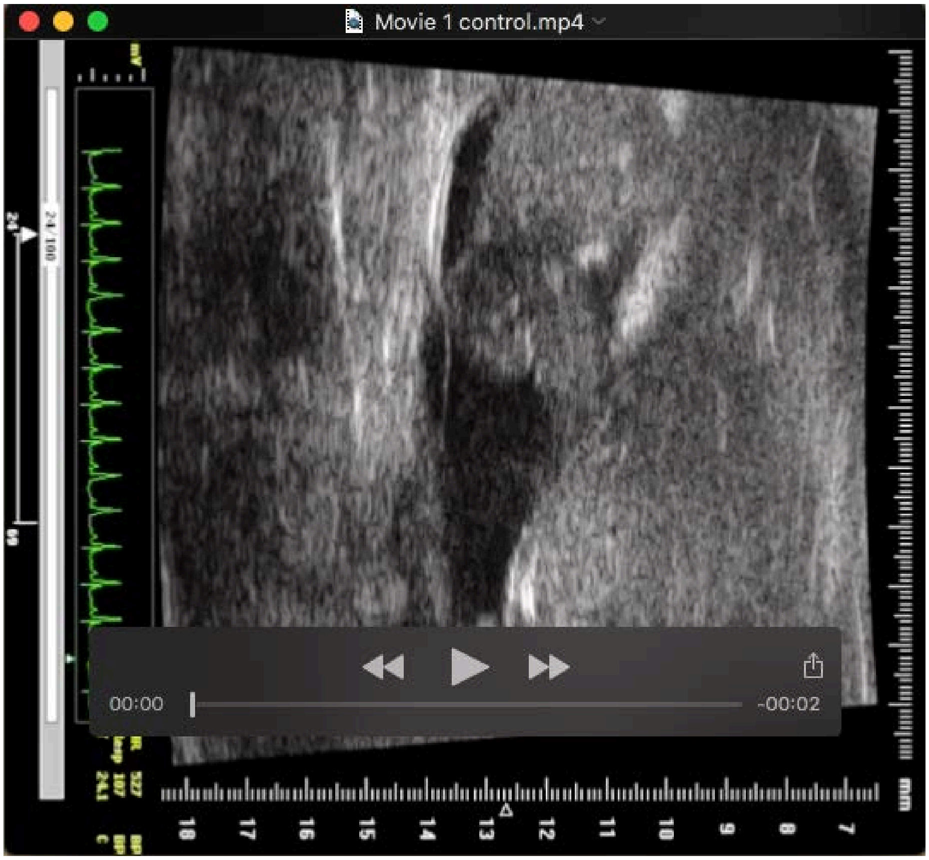

Movie 1. *In utero* ultrasound of *Rdh10*$^{+/+}$ control embryos reveals mouth opening and tongue withdrawal accompany backwards head extension.

Disease Models & Mechanisms • Supplementary information

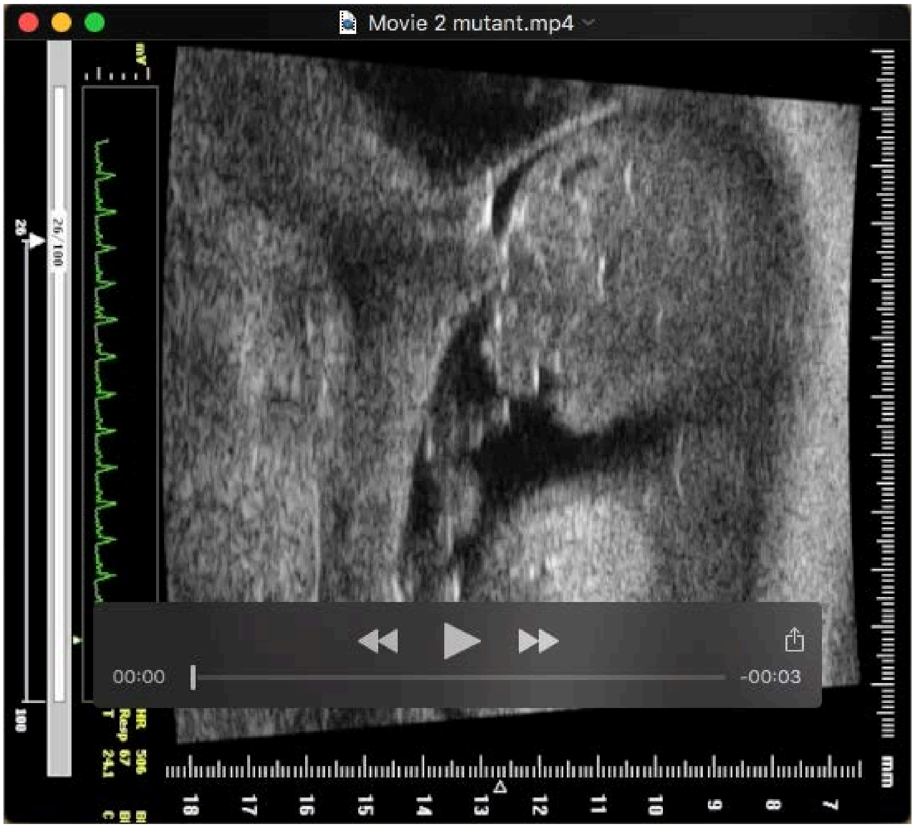

Movie 2. *In utero* ultrasound of *Rdh10*$^{flox/flox}$ mutant embryos reveals backwards head extension only without mouth movement.
